# Inflammatory models of depression in rodents and humans

**DOI:** 10.3389/fpsyt.2026.1687579

**Published:** 2026-02-02

**Authors:** Dmitrii D. Markov, Svetlana A. Zozulya, Oleg V. Dolotov

**Affiliations:** 1 Lopukhin Federal Research and Clinical Center of Physical-Chemical Medicine of Federal Medical Biological Agency, Moscow, Russia; 2 National Research Center “Kurchatov Institute”, Moscow, Russia; 3 Mental Health Research Center, Moscow, Russia; 4 Faculty of Biology, Lomonosov Moscow State University, Moscow, Russia

**Keywords:** depression, inflammation, LPS, models, proinflammatory cytokines

## Abstract

The precise pathophysiological mechanisms underlying major depressive disorder (MDD) remain poorly understood. Substantial evidence implicates immune-mediated mechanisms in the pathogenesis of this clinically heterogeneous and multifactorial disease. This review provides a comprehensive synthesis of current knowledge regarding the association between inflammation and depression, critically evaluates established approaches for modeling inflammation-induced depressive states in both rodents and humans, and assesses these models against standard validity criteria. The empirical link between depression and immune dysregulation is supported by several key lines of evidence: elevated circulating cytokine levels in MDD patients, the induction of depressive symptoms during therapeutic administration of pro-inflammatory cytokines, the significant comorbidity of MDD with chronic inflammatory diseases, the anti-inflammatory properties of conventional antidepressants and the alleviation of depressive symptoms during anti-inflammatory therapy. Various immune activators are employed to model inflammation-associated depression. Experimental human models primarily utilize lipopolysaccharide (LPS) administration or typhoid vaccination. Corresponding rodent models employ LPS, direct administration of pro-inflammatory cytokines, or immunization with BCG vaccine. In rodent models, the administration of classical antidepressants effectively attenuates the severity of depressive-like behaviors induced by LPS. The predictive validity of the LPS-induced depression model is further corroborated by the demonstrated antidepressant-like efficacy of the rapid-acting agent ketamine. Data on the effects of antidepressants within controlled experimental inflammatory models in humans remain scarce and the impact of novel rapid-acting agents like ketamine and psychedelics in this context remains entirely unexplored. Human experimental studies demonstrate high consistency and reproducibility regarding LPS dosing, experimental timelines, and symptom assessment. Conversely, rodent studies exhibit significant heterogeneity across these same parameters. A major limitation shared by most existing inflammatory models, in both humans and rodents, is their non-chronic nature and the development of tolerance with repeated inducer administration. A critical translational challenge lies in establishing the homology between behavioral outcomes in rodents and the clinical symptomatology of human depression. The development of refined inflammatory models of depression that more rigorously satisfy established validity criteria is imperative. Such models are crucial for elucidating the underlying pathophysiological mechanisms of the disorder and for facilitating the discovery of novel, effective pharmacotherapies.

## Highlights

- Inflammation is a significant factor in inducing a depressive-like state.
- Inflammatory models rely on administering immune system activators.
- Discrepancies exist between human and rodent inflammatory models of depression.
- LPS doses differ by 100,000–1,000,000 times between humans and rodents.
- Limited correspondence exists between human depressive symptoms and rodent depressive-like behaviors.

## 1 Introduction

The precise etiological causes and underlying pathophysiological mechanisms of major depressive disorder (MDD, depression) remain inadequately elucidated, despite extensive scientific investigation into this highly prevalent mental illness. This persistent knowledge gap is attributable, in part, to the inherent complexity, clinical heterogeneity, and profoundly multifactorial nature of the disorder. A further, significant impediment to research progress is the considerable challenge of faithfully replicating core symptomatic features of human depression, most fundamentally, sustained low mood, in preclinical animal models. This limitation substantially hinders both the advancement of pathophysiological understanding and the discovery and development of novel therapeutic interventions, for which there is a pressing clinical need. Contemporary animal models are, therefore, predominantly predicated on specific etiological hypotheses describing putative triggers for the disorder. Consequently, given the well-established role of stress as a major risk factor for depression, paradigms such as chronic unpredictable mild stress (CUMS) and social defeat stress are extensively employed to model stress-associated depressive states (1, 2).

Inflammation represents another critical etiological factor implicated in the pathogenesis of depression (3–5). The activation of the immune system has been demonstrated to induce a depressive-like state in both animal models and humans. For instance, upper respiratory tract infections in humans can affect mood and cognition (6), while exposure to an influenza vaccine has been shown to alter neural reward processing (7). Acting in concert with stress, inflammation exerts a significant influence on the reward circuitry in both humans and animals, and is strongly associated with the induction of anhedonia (8). This symptom, the reduced capacity to experience pleasure, alongside persistent low mood, constitutes a core diagnostic criterion for major depressive disorder in humans. The constellation of mood and behavioral alterations provoked by immune activation bears a direct resemblance to key depressive symptomatology (9).

Animal models of depression are an indispensable tool for investigating the pathophysiological mechanisms underlying this disorder and for the preclinical development of novel antidepressant drugs. To model inflammation-associated depression, researchers commonly administer various inflammatory inducers to animals, with lipopolysaccharide (LPS), a primary component of the outer membrane of gram-negative bacteria, being the most widely utilized agent (10). According to established validation criteria for animal models, as outlined by P. Willner (11), an animal model of depression should satisfy three key validities: face validity (phenomenological similarity to the human condition), construct validity (shared etiology and pathogenesis), and predictive validity (sensitivity to known therapeutic interventions). Inflammatory models of depression offer a unique advantage by allowing for direct comparison of results obtained under controlled experimental conditions in both human participants and rodents. This review aims to synthesize existing data on inflammatory models of depression in rodents and humans and to evaluate these models through the lens of the aforementioned validity criteria. We will discuss the advantages and limitations of these models, address challenges in their implementation, and analyze the parallels and discrepancies between LPS-induced depressive states in humans and rodents.

## 2 Construct validity, depression and inflammation

### 2.1 Sickness behavior

The clinical presentation of infectious disease typically includes non-specific systemic symptoms such as malaise, fever, headache, weakness, fatigue, and loss of appetite. These symptoms are attributable to elevated circulating cytokine levels consequent to immune system activation. In 1988, Hart B.L. posited that this constellation of behavioral responses to infection constitutes a highly organized, adaptive strategy evolved to enhance organismal survival within a natural habitat (12). This state, termed sickness behavior, is characterized by a coordinated reduction in locomotor activity, food intake, social interaction, and sexual behavior. The phenotypic manifestations of sickness behavior bear a notable resemblance to the symptoms of human depression, with shared features including low mood, fatigue, and significant appetite alterations. This symptomatic overlap suggests the potential involvement of common pathophysiological pathways linking depression and the systemic inflammatory response to infection. Furthermore, this behavioral similarity is not the sole indicator implicating immune system dysregulation in the etiology of depression.

### 2.2 Increased levels of inflammatory markers in depression

Major Depressive Disorder is frequently associated with a measurable elevation of inflammatory mediators in peripheral blood (13). Compared to healthy controls, patients with depression exhibit significantly higher circulating levels of key pro-inflammatory cytokines and acute-phase proteins, including IL-1, IL-6, IL-10, TNF-α and CRP (14). Dahl et al. documented significantly elevated levels of 9 cytokines (IL-1β, IL-1RA, IL-5, IL-6, IL-7, IL-8, IL-10, G-CSF, IFN-γ) in depressed patients (15). Similarly, He et al. reported increased concentrations of 13 cytokines, chemokines, and growth factors, including IL-1β, IL-6, IL-17A, IL-1RA, MCP-1, Eotaxin, IL-8, FGF, IL-7, VEGF, IL-2, IL-4, IL-5 (16). A more comprehensive analysis by Xu et al. found that among 37 measured serum proteins, 15 were upregulated (IL-1β, IL-2, IL-4, IL-6, IL-8, IL-10, IL-12, IL-13, IL-15, CCL3, CCL4, CCL11, TNF-α, FGF basic, TSLP) while 8 were downregulated (IL-5, IL-12 p70, IL-16, CCL17, CXCL10, VEGF-C, TNF-β, PIGF) in MDD patients compared to controls (17). A broader meta-analysis corroborates elevated levels of CRP, IL-3, IL-6, IL-12, IL-18, sIL-2R and TNF-α in depression (18). A meta-analysis focusing on drug-naïve adolescent populations revealed a significant association between TNF-α and MDD when compared to healthy controls. However, other measured peripheral blood cytokines, including IL-1β, IL-4, IL-6, IL-8 and IFN-γ did not demonstrate a significant correlation with MDD (19). The baseline concentrations of inflammatory markers demonstrate correlation with the severity of specific symptoms, rather than with the overall depression severity (20). Interestingly, inflammatory marker levels are negatively correlated with the severity of mood and anxiety symptoms, yet exhibit both negative and positive correlations with neurovegetative symptoms, such as alterations in appetite, weight, sleep, and libido (20).

Evidence suggests a significant association between elevated concentrations of peripheral inflammatory markers and an increased risk of suicidal behavior in patients diagnosed with MDD. MDD patients at higher suicide risk present with elevated levels of IL-6, CRP, TNF-α, CXCL-2 and IFN-γ, alongside reduced IL-2 and IL-8 (21). However, this finding is not universal, as other research reports that a higher plasma IL-2 level is associated with increased suicidal ideation in first-episode, drug-naïve MDD patients (22), highlighting the complexity and heterogeneity of the inflammatory response in depression.

An elevated serum level of CRP serves as a key clinical biomarker of systemic inflammation. Comparative analyses reveal distinct CRP concentrations across clinical populations: healthy volunteers present a mean level of 1.3 mg/L, whereas untreated patients with MDD exhibit a significantly higher concentration of 2.50 mg/L. Furthermore, this inflammatory marker demonstrates variation with treatment response, with levels averaging 2.1 mg/L in treatment-responsive MDD patients and rising to 3.1 mg/L in those with treatment-resistant depression (23). It is noteworthy that the magnitude of difference between depressed patients and control subjects is not always pronounced, though it remains statistically significant, as illustrated by a comparison of 2.4 mg/L versus 2.1 mg/L, respectively (24). CRP level is specifically correlated with fatigue, a neurovegetative symptom, but not with other symptoms such as anhedonia, sleep disturbances, or appetite changes, indicating that this inflammatory marker is linked to a specific subset of inflammation-related depressive features (25). However, these data were obtained in a nationally representative community study and may not be fully applicable to patients with clinically confirmed MDD. Some authors use CRP levels to prove the association between inflammation and clinical subtypes of depression identified within the heterogeneous diagnostic construct of MDD (26). CRP levels were shown to be higher in patients with atypical depression compared to patients with melancholic depression, patients with MDD, and healthy individuals (27). In the study (28), patients diagnosed with MDD according to DSM-V were divided into five clinical subtypes: atypical, anxious, melancholic, psychotic, and MDD not otherwise specified. The authors found a statistically significant increase in CRP levels among patients with atypical depression compared to other subtypes. Logistic regression analysis showed that significant predictors associated with high CRP levels were suicidal behavior and atypical depression, highlighting the contribution of different pathophysiological mechanisms to the formation of different clinical subtypes of depression. It was found that patients with “atypical” major depressive disorder, characterized by autonomic symptoms, hypersomnia, and hyperphagia, were approximately two times more likely to have higher CRP levels than healthy controls or patients with “typical” major depressive disorder, characterized by depressed mood, sleep disturbance, and decreased appetite (29).

The American Heart Association stratifies cardiovascular disease risk based on CRP levels, classifying a concentration below 1.0 mg/L as low risk, 1.0 to 3.0 mg/L as moderate risk, and above 3.0 mg/L as high risk (30). These established thresholds are frequently adopted within psychiatric research to quantify the degree of systemic inflammation in patients with depression. Clinically significant systemic inflammation, defined by a CRP concentration exceeding 3 mg/L, is present in only 21–27% of patients with depression (24, 31, 32) and in 7.4–16.8% of healthy individuals (23, 24). Elevated CRP is clinically significant, being associated with suicidal behavior in depressive patients (33) and potentially serving as a predictive biomarker for future depression onset (34–36), underscoring the utility of its assessment. While the existing CRP criteria are explicitly designed for cardiovascular risk stratification, the development of analogous criteria for affective disorders could enable a more precise stratification of patients into inflammatory subtypes, thereby refining both experimental models and therapeutic approaches. However, when interpreting data on CRP levels in MDD, it is likely that it should be taken into account that the tests measure the pentameric form of CRP, which has weak anti-inflammatory activity, while the monomeric form of CRP exhibits pro-inflammatory properties and its levels may be associated with the severity of symptoms and resistance to treatment in MDD (37).

Researchers frequently assess the levels of both pro-inflammatory and anti-inflammatory cytokines in the context of depression. However, reported concentrations of pro-inflammatory cytokines in patients with depression can vary significantly across different studies (see **Table 1**). Consequently, a more methodologically sound approach is to compare the difference in cytokine levels between healthy controls and depressed patients within the same study. For instance, within-study comparisons demonstrate that patients with depression exhibit nearly a two-fold increase in blood IL-1β and a greater than two-fold increase in TNF-α relative to healthy controls (38). The magnitude of pro-inflammatory cytokine elevation in depressive patients typically corresponds to a 1.4 to 2-fold increase relative to levels quantified in healthy controls (see **Table 1**). The cytokine profile in depression is further modulated by sex and clinical subtype. Sex-stratified analyses indicate that levels of CRP and IL-6 are elevated specifically in depressed females compared to healthy controls, an effect not observed in males. In contrast, TNF-α is elevated in depressed individuals irrespective of sex (39). Regarding clinical presentation, the prevalence of low-grade inflammation is higher in atypical depression (34.8%) than in MDD without atypical features (26.8%) (40). Furthermore, while patients with both melancholic and non-melancholic depression exhibit increased CRP and decreased IFN-γ compared to healthy individuals, elevated levels of IL-12 and IL-10 appear specific to the melancholic subtype (41). Collectively, these findings suggest that the observed pattern of cytokine alterations is not uniform but is significantly influenced by patient heterogeneity, including the sex ratio of the cohort and the distribution of depression subtypes within the study sample.

**Table 1:** Cytokine levels in MDD patients and healthy subjects.

Participants	IL-1β, pg/ml	IL-6, pg/ml	TNF-α, pg/ml	IL-1RA, pg/ml	IL-10, pg/ml	References
Healthy subjects	4.17 ± 2.42	1.30 ± 0.53	25.10 ± 4.94	109.66 ± 54.58	8.62 ± 7.97	(16)
MDD	6.15 ± 3.60*	1.89 ± 0.97*	27.88 ± 5.34	149.39 ± 38.75*	11.30 ± 5.90	
Healthy subjects	6.26 ± 0.17	1.60 ± 0.20	2.67 ± 0.11	872.01 ± 85.30	1.94 ± 0.21	(17)
MDD	9.48 ± 1.07*	11.42 ± 2.26*	4.63 ± 0.39*	1098.87 ± 142.14	3.16 ± 0.41*	
Healthy subjects	5.99 ± 2.31	not determined	28.22 ± 9.96	not determined	not determined	(38)
MDD	11.21 ± 8.46*	not determined	63.37 ± 49.64*	not determined	not determined	
Healthy subjects	0.44 (0.28—0.95)	2.35 (1.28—6.28)	9.63 (4.92—22.65)	41.29 (23.89—82.07)	2.49 (0.97—6.03)	(15)
MDD	0.83 (0.48—1.48) *	5.21 (2.74—7.67) *	16.99 (9.36—29.77)	56.93 (39.39—104.43) *	4.32 (2.29—5.61) *	
Healthy subjects	6.39 ± 0.94	1.52 (1.08, 2.05)	2.88 ± 0.87	not determined	not determined	(316)
MDD	7.55 ± 2.49	4.31 (2.20, 13.77) *	4.50 ± 2.43*	not determined	not determined	

*Significant differences, P value < 0.05.

The relatively modest magnitude of cytokine elevation observed in patients with depression compared to healthy controls is indicative of a low-grade systemic inflammatory state. In conditions such as sepsis, TNF-α levels can be elevated by an order of magnitude (approximately 10-fold) relative to healthy individuals (42). It is evident that elevated pro-inflammatory cytokines do not serve as a specific biomarker for depression, given that increased inflammatory markers are a common feature across a wide spectrum of somatic and psychiatric disorders. Furthermore, substantial variability in inflammatory marker levels exists within both healthy and depressed populations (see **Table 1**). This heterogeneity necessitates a critical, yet unresolved, diagnostic question: what quantitative thresholds of cytokines or acute-phase proteins definitively delineate a healthy state from a state of clinically significant low-grade inflammation?

Thus, significant immune system activation is not a universal feature of depression, as only a subset of patients exhibits elevated inflammatory markers. The field currently lacks clear diagnostic criteria to define low-grade inflammation within the context of mental disorders. Consequently, the relatively small increase in cytokine levels observed across a depressed cohort may largely reflect the heterogeneity of the sample. Elevated levels in a proportion of patients may be diluted by normal levels in others, thereby attenuating the mean difference when compared to healthy controls.

Different levels of inflammatory biomarkers in patients with MDD may correspond to the presence of several underlying biological mechanisms associated with different genetic predispositions, i.e. endophenotypes of the disease, which confirms the heterogeneity of depression and allowing the identification of subgroups of patients regardless of the phenomenological manifestations of MDD (43). It is proposed to divide MDD into subtypes based on markers of dysregulation of three neurobiological systems: the dopamine system, the hypothalamic-pituitary-adrenal (HPA) axis, and markers of chronic inflammation (44). One such marker may be CRP: patients with treatment-resistant depression have the highest CRP levels compared to both treatment-responsive and treatment-naive patients (23). Elevated CRP levels and treatment resistance were also associated with other aspects of clinical depression, including obesity, autonomic symptoms, fatigue and sleep disturbances, state anxiety, and adverse childhood history. The authors propose the existence of a clinically and immunologically diagnosable subsyndrome of “inflammatory depression”, encompassing patients with MDD, who are likely to benefit from second-line treatment with anti-inflammatory agents (23).

Arguments in favor of the existence of an “inflammatory subtype” of depression link the presence of a corresponding biological profile not only with resistance to therapy, but also with the presence of depression with pronounced somatic and neurovegetative (sleep disturbance, appetite, decreased libido), affective (including depressed mood, suicidal thoughts and irritability) and cognitive symptoms (including affective bias and guilt) (45). It was found that higher CRP levels were associated not only with neurovegetative symptoms, but also with symptoms of depressed mood, changes in appetite and sleep problems, and anxiety, while higher IL-6 levels were associated with anhedonia (46).

The findings of distinct cytokine and CRP profiles in clinical subtypes of MDD and the association of different markers with different symptoms suggest that different clinical manifestations of depression (phenotypes) may be mediated by different inflammatory pathways and have different endophenotypes. This consideration is consistent with the approach proposed by the Research Domain Criteria (RDoC) program of the National Institute of Mental Health (NIMH). The RDoC program complements the current classification of mental disorders (DSM-5) and proposes a multi-level analysis of psychopathological disorders based on the identification of various biomarkers (electroencephalography, neuroimaging, cognitive, behavioral, molecular, and biochemical) associated with the disease through common genetic mechanisms (47). The phenotype of depression can be formed as a result of a complex interaction of genetic and epigenetic factors, family history, early developmental characteristics, the influence of stress, and other factors. Application of the RDoC approach to the analysis of the association between elevated CRP levels in subjects without a clinical diagnosis of MDD, the presence of fatigue syndrome, and the development of depression (46) may allow to identify several endophenotypes characterized by similar biological profiles but differing in clinical manifestations (both transnosological and specific to this disorder): a depressive endophenotype, an endophenotype with increased vulnerability to the development of depression, and an endophenotype not associated with depression. The use of the RDoC paradigm in patients with clinically confirmed MDD can help identify the features of psychopathological symptoms among individuals with the same phenotypic manifestations of depressive disorders, identify subjects with different inflammatory profiles, which is important for clarifying the mechanisms of the inflammatory response in the development of depression, predicting the course of MDD, and personalizing patient therapy (48).

Thus, a proportion of patients with MDD exhibit elevated levels of inflammatory markers, and certain inflammatory profiles may be associated with symptoms, their severity, and treatment resistance. The heterogeneity of the “inflammatory subtype” of depression itself suggests the presence of various mechanisms leading to elevated inflammatory markers and the manifestation of MDD symptoms. On the other hand, the heterogeneity of inflammatory profiles in depression may be due to the fact that inflammatory biomarker levels are influenced by a wide range of factors, such as obesity, diet, exercise, smoking, age, gender, race, socioeconomic status, and medication use (49). These factors are associated with both inflammatory biomarker profiles and the development of depression. Moreover, inflammatory profiles in depression may depend on the stage of depressive symptom development (50). Therefore, providing detailed background information on the aforementioned factors, the duration of depressive episodes, and the presence or absence of treatment for depression and comorbidities will be important in studies examining the associations of inflammatory biomarker profiles with depressive symptoms and their severity. An additional significant source of heterogeneity arises from methodological variation. To mitigate this, cohort formation should be guided by stringent, highly consistent inclusion criteria, as greater baseline homogeneity among participants predicts more uniform experimental outcomes. Furthermore, standardization should extend to all measurement tools, including the diagnostic scales and questionnaires used to assess depression severity, as well as the protocols for biological sample handling and analytical assay kits.

### 2.3 Proinflammatory cytokine therapy

The induction of depressive symptoms in patients undergoing cytokine therapy provides compelling clinical evidence for a causal role of inflammation in the pathogenesis of depression. For instance, until the advent of direct-acting antivirals, the combination of ribavirin and IFN-α constituted the standard therapeutic regimen for patients with chronic hepatitis C. A significant adverse effect of this treatment was the induction of depressive symptoms following chronic IFN-α administration. The reported incidence of depression in this patient population varies considerably, with estimates ranging from 23% to 40.7% (51–56) across studies, and a meta-analysis indicating a development rate of approximately 25% within 24 weeks of initiating therapy (57). The onset of depressive symptoms in these patients is linked to a therapy-induced increase in pro-inflammatory cytokine levels (58). Specifically, IFN-α administration elevates circulating concentrations of IL-6, IL-8, and IL-10 with depressive symptoms typically emerging within 2 to 4 weeks of treatment initiation (59). Notably, IFN-α-induced depression during hepatitis C therapy is characterized predominantly by somatic symptomatology, including loss of appetite, insomnia, fatigue, psychomotor retardation, headaches, backaches, muscle aches, and gastrointestinal symptoms, rather than by core affective symptoms such as low mood or anxiety, particularly in cases of early onset within the first six weeks of treatment (53).

Similarly, treatment for malignant melanoma with IFN-α is associated with a significant incidence of depressive symptomatology (60). Within a 12-week treatment period, depression develops in approximately 45% of these patients (61). The onset of symptoms follows a distinct temporal pattern: neurovegetative and somatic symptoms emerge as early as two weeks after initiating IFN-α therapy. In contrast, depressed mood, anxiety, and cognitive dysfunction typically manifest later, after 8 to 12 weeks of treatment. While neurovegetative and somatic symptoms manifest non-specifically in a substantial proportion of patients, the emergence of depressed mood, anxiety, and cognitive dysfunction occurs predominantly in those who subsequently meet the diagnostic criteria for a MDD as defined by the DSM-V. Fatigue or loss of energy is observed in up to 80% of patients undergoing IFN-α therapy, depressed mood in 60%, anhedonia in 30%, and suicidal ideation in 10% (62).

The development of depressive symptoms following IFN-α administration in humans reflects an independent psychiatric consequence of sustained cytokine elevation, irrespective of the underlying somatic pathology. This causal relationship is substantiated by experimental studies in non-human primates, where chronic central or peripheral IFN-α administration reliably induces depressive-like behavior, including anhedonia (63–66).

The data provide robust clinical and experimental evidence that chronically elevated cytokine levels is a direct, independent causal factor in the pathogenesis of depression, independent of underlying medical illness.

### 2.4 Comorbidity of depression with inflammatory diseases

Further evidence supporting the involvement of inflammation in the pathogenesis of depression is the well-documented comorbidity between this affective disorder and chronic inflammatory diseases. For instance, the incidence of depression in multiple sclerosis ranges from 24% to 31% (67–69). Among patients with rheumatoid arthritis, the prevalence of a depressive disorder is approximately 17% (70), while depressive symptoms are observed in 28–55% of this population (71). Meta-analyses indicate that the incidence of major depressive disorder in patients with inflammatory bowel disease is approximately 25% (72), and depressive symptoms are present in roughly 28% of individuals with psoriasis (73). This pattern indicates that approximately one-quarter of patients with systemic inflammatory conditions exhibit significant depressive symptomatology. This consistent comorbidity across diverse inflammatory conditions supports the hypothesis that shared inflammatory pathways contribute to the development of depression. However, as depression does not manifest in all affected individuals, the potential contribution of additional mechanisms, such as those related to the psychological stress of chronic illness, cannot be excluded.

### 2.5 Anti-inflammatory therapy for the treatment of depression

Elevated baseline levels of inflammatory markers in patients with depression have been associated with a diminished response to first-line antidepressant pharmacotherapy (74). If immune system activation is integral to the pathophysiology of depression, then pharmacological agents with anti-inflammatory properties may possess therapeutic efficacy. Consequently, the efficacy of several such compounds has been evaluated (75). For instance, the COX-2 inhibitor celecoxib has demonstrated efficacy in postpartum depression, with treated patients showing greater symptomatic improvement and lower levels of IL-6, CRP, and TNF-α (76). Celecoxib appears most effective as an adjunctive therapy, enhancing the antidepressant action of agents like fluoxetine (77) or reboxetine (78). Similarly, combining selective serotonin reuptake inhibitor (SSRI) therapy with acetylsalicylic acid has been shown to reduce depressive symptoms in patients who were previously non-responsive to SSRI monotherapy (79). The meta-analysis further indicates that most pharmacological agents with anti-inflammatory properties, including non-steroidal anti-inflammatory drugs (NSAIDs), omega-3 polyunsaturated fatty acids, statins, glucocorticoids, cytokine inhibitors, and minocycline, are effective only when used in combination with antidepressant medications (80, 81). Among the various anti-inflammatory agents investigated, corticosteroids demonstrate superior efficacy in alleviating depressive symptoms (82). Notably, the interaction is not uniformly positive, as some evidence suggests NSAIDs may potentially antagonize the therapeutic efficacy of SSRIs (83). At present, robust evidence supporting the efficacy of anti-inflammatory drug monotherapy for major depression is lacking. This limited efficacy may be partly attributable to the marked clinical heterogeneity of MDD. The therapeutic effect of an anti-inflammatory agent is likely restricted to the inflammatory subtype characterized by elevated pro-inflammatory cytokines. Consequently, its clinical benefit may be obscured within a broader, non-stratified patient cohort, where a substantial proportion of individuals do not exhibit significant peripheral inflammation. Reflecting this complexity, a recent meta-analysis concluded that no clear clinical recommendations can yet be formulated regarding anti-inflammatory treatment for MDD (84).

The stratification of patients with depression by baseline CRP levels prior to LPS administration revealed that participants with higher baseline CRP exhibited elevated IL-6 responses and significantly greater increases in self-reported anhedonia (85). This indicates that depressed patients with a pre-existing state of elevated systemic inflammation possess a heightened vulnerability to developing anhedonia, such as during infection or exacerbation of inflammatory disease. Unfortunately, no longitudinal clinical study in patients with depression has yet demonstrated the clinical utility of stratifying patients based on inflammatory status. Consequently, it remains unclear whether such stratification can identify individuals who would benefit from adjunctive anti-inflammatory therapy to improve treatment outcomes.

A significant comorbidity exists between certain inflammatory diseases and the development of depression. Several immunobiological agents, monoclonal antibodies targeting specific cytokines or their receptors, are now approved for the treatment of these underlying inflammatory conditions. For several such diseases, these therapies have demonstrated efficacy in alleviating co-occurring depressive symptoms, albeit with variable success. Antibodies targeting IL-6 and IL-12/23 demonstrate a significant effect in alleviating depressive symptoms, whereas therapies targeting TNF-α exhibit inconsistent efficacy (86). For instance, Ustekinumab (an IL-12/23 antibody) significantly improves symptoms of anxiety and depression in patients with psoriasis (87, 88). Similarly, treatment with Secukinumab (an IL-17A antibody) results in improvement of anxiety and depressive symptoms in psoriatic patients (89). The TNF-α inhibitor etanercept (90) and adalimumab (TNF-α antibody) (91) have also been associated with a reduction in depressive symptoms in patients with psoriasis. Furthermore, sirukumab and siltuximab (IL-6 antibodies) improve depressive symptoms in patients with rheumatoid arthritis and multicentric Castleman’s disease (92). Critically, the efficacy of these anti-cytokine therapies appears to be context-dependent. They are not consistently effective in treating depressive states that are not comorbid with a primary inflammatory somatic disease. For example, infliximab (a TNF-α antibody) did not significantly reduce depressive symptoms in patients with bipolar depression (93) and failed to improve symptoms in patients with treatment-resistant depression (94). A central unresolved question pertains to the mechanism of symptom alleviation during anti-cytokine therapy. It remains unclear whether clinical improvement results from the direct pharmacological blockade of specific cytokine-mediated pathways or is a secondary consequence of the amelioration of the primary somatic disease.

A further unresolved issue concerns the role of the blood-brain barrier (BBB) in mediating the antidepressant effects of anti-inflammatory agents. It remains unclear whether the clinical efficacy of these drugs in alleviating depressive symptoms is contingent upon their ability to cross the BBB and exert direct central actions. This raises a critical question regarding the efficacy of anti-inflammatory drugs with limited blood-brain barrier permeability. If neuroinflammation is a primary pathogenic mechanism in depression, what is the therapeutic potential of drugs that do not cross the BBB and therefore cannot directly access the central nervous system? Will anti-inflammatory drugs that cross the blood-brain barrier exert a different effect on depressive symptoms than those whose action is confined to the periphery? To resolve these questions, it is imperative to conduct randomized clinical trials that directly compare the antidepressant efficacy of anti-inflammatory drugs with established BBB permeability profiles. Such studies necessitate a collaborative framework involving specialists in pharmacokinetics, pharmacodynamics, and pharmacogenetics to ensure accurate drug characterization and interpretable results. The findings from these trials will delineate the relative contributions of central neuroinflammation versus peripheral inflammation to the pathophysiology of depressive disorder.

The accumulated evidence suggests potential clinical applications, which can be preliminarily summarized as follows: 1) For patients with depression, particularly in cases of treatment resistance or comorbid inflammatory conditions consider measuring peripheral inflammatory markers which elevated levels can serve as a prognostic indicator for potential poor response to first-line antidepressants and as a biomarker of depression associated with inflammation. 2) For patients with elevated inflammatory markers or comorbid inflammatory disease, consider adding an anti-inflammatory drugs as adjunctive therapy to standard antidepressant treatment. 3) For patients with depression secondary to somatic inflammatory diseases (e.g., psoriasis, rheumatoid arthritis), treatment with specific immunobiologicals can lead to significant improvement in depressive symptoms. This underscores the importance of collaboration between psychiatrists, rheumatologists, dermatologists and other clinical specialists to optimize both somatic and psychiatric outcomes. 4) Clinical observation of depressive symptom trajectories in patients undergoing targeted anti-cytokine therapies for primary inflammatory diseases represents a valuable translational research opportunity. Systematic assessment in this population can help identify new potential targets and treatment options.

### 2.6 Increased intestinal permeability

The precise mechanisms underlying the elevated levels of pro-inflammatory cytokines observed in depression remain poorly understood. One proposed factor contributing to immune system activation in depressed patients is the increased translocation of bacterial products, particularly from the intestinal lumen, into the systemic circulation. The influence of the gut microbiome and associated intestinal permeability on neuronal and glial function, and its consequent role in the pathogenesis of affective disorders, constitutes an active area of investigation (95, 96). Evidence for increased translocation of bacterial products from the intestinal lumen into the systemic circulation in depression is provided by the elevated blood levels of IgM and IgA specific to antigens of gram-negative bacteria observed in depressed patients (97, 98). Furthermore, plasma levels of lipopolysaccharide-binding protein (LBP) correlate with the lactulose/mannitol ratio, a validated clinical marker of intestinal permeability (99).

Depressed patients exhibit significantly elevated plasma concentrations of several biomarkers linked to altered gut barrier integrity, including LPS, FABP2/I-FABP, zonulin, and claudin-5, compared to healthy controls (100, 101). Increased LBP levels and elevated LBP/sCD14 ratios in women have been associated with a greater likelihood of developing depressive symptoms in the future (102).

However, the evidence for altered intestinal permeability in depression is not unequivocal. Findings regarding specific biomarkers remain inconsistent and often context-dependent. For instance, while patients with a recent suicide attempt exhibit elevated levels of FABP2/I-FABP that correlate with depressive symptom severity, they simultaneously show lower levels of zonulin. In contrast, no significant differences in zonulin or FABP2/I-FABP levels were detected in healthy controls and patients with MDD who had no history of suicide attempts. Furthermore, that same study found no difference in sCD14 levels between depressed patients and controls (103). Similarly, in patients with inflammatory bowel disease, depressive symptoms correlate positively with levels of calprotectin and LBP but show no association with zonulin or FABP2/I-FABP (104). Adding to the complexity, one study found no correlation between the established lactulose/mannitol ratio and levels of FABP2/I-FABP or zonulin (99). These inconsistencies suggest that the utility of these specific proteins as reliable, standalone markers of intestinal permeability in depression remains unresolved.

In summary, the currently available evidence in this area remains both limited and inconsistent, precluding any definitive conclusion regarding a causal relationship between increased intestinal permeability and the pathogenesis of depression.

The well-documented comorbidity of depression with inflammatory diseases, coupled with the induction of depressive symptoms during chronic cytokine administration, suggests that sustained systemic inflammation can function as a causal factor in the development of depression. Conversely, the observation of elevated pro-inflammatory cytokines in a subset of depressed patients raises the alternative possibility that the depressive state itself, or the chronic stress that frequently precedes it, may trigger immune system activation. The mechanisms underpinning this potential stress-induced immune system activation remain incompletely elucidated. One prominent hypothesis implicates hyperactivation of the HPAA and elevated cortisol levels in inducing a state of glucocorticoid resistance within both central nervous system and immune cells (105). This resistance disrupts the negative feedback self-regulation of the HPAA and concurrently diminishes the efficacy of endogenous glucocorticoids in suppressing inflammatory responses. Stress-related increases in peripheral cytokine levels may also be mediated by alternative pathways, such as compromised intestinal barrier function. Empirical evidence indicates that diverse stressors can compromise intestinal barrier integrity in both human and rodents. For instance, rats subjected to electric foot shock exhibit enhanced translocation of LPS across the intestinal wall, concomitant with a rise in plasma IL-6 (106). In humans, acute psychological stress (e.g., public speaking) increases intestinal permeability, an effect associated with HPAA and mast cell activation (107). Furthermore, individuals experiencing hostile marital interactions, a potent source of chronic psychosocial stress, exhibit elevated levels of LBP and higher LBP/sCD14 ratios (108). Evidence suggests that stress may increase intestinal permeability, allowing bacterial products to enter the bloodstream. The “leaky gut” hypothesis offers a plausible explanation for how psychological stress translates into inflammatory response, but more consistent research is needed to confirm it as a definitive cause.

### 2.7 Peripheral and central inflammation

The development of MDD is increasingly understood as a process involving bidirectional communication between peripheral inflammation and central neuroimmune activation. Systemic inflammation and functional alterations in peripheral immune organs, such as the gut, liver, spleen, and adipose tissue, can modulate brain activity. Within the CNS, activated microglia and astrocytes drive neuroinflammation, which disrupts neurotransmission, impairs neuroplasticity, and can lead to neuronal damage. These peripheral and central immune systems are linked via neural and humoral pathways, collectively contributing to the development and progression of MDD (109).

Systemic inflammation can influence central nervous system function and precipitate depressive symptoms through multiple pathways. One primary route involves the long-term disruption of the BBB by peripheral inflammatory mediators, leading to microglial activation (110). Additional communication pathways exist (111). These include the induction of prostaglandin synthesis in BBB endothelial cells, signaling via the vagus nerve, access through circumventricular organs, and the direct infiltration of immune cells into the brain.

Activated microglia initiate a pathological cascade characterized by elevated glutamate and cytokine concentrations, reduced neurotrophin levels, and increased oxidative stress (110), all of which disrupt neuronal homeostasis. Furthermore, proinflammatory cytokines induce indoleamine 2,3-dioxygenase (IDO), a key enzyme in the tryptophan catabolic pathway. IDO catalyzes the conversion of tryptophan to kynurenine, which is subsequently metabolized to quinolinic acid, a neurotoxic NMDA receptor agonist. Concurrently, proinflammatory cytokines deplete tetrahydrobiopterin, an essential cofactor for synthesizing serotonin, dopamine, and norepinephrine (112). Inflammatory response can affect metabolic and molecular pathways influencing neurotransmitter systems that can ultimately affect neurocircuits that alter behavior, thereby contributing to the manifestation of depressive symptomatology (112).

Due to ethical constraints, proinflammatory cytokine levels in humans are measured almost exclusively in peripheral blood, occasionally in CSF, and within the brain only postmortem. This limitation impedes the direct assessment of how central neuroinflammation relates to the severity of peripheral inflammation in depression. Consequently, existing data do not yet provide a clear understanding of the correlation between peripheral inflammatory markers and neuroinflammation. Evidence from a meta-analysis of postmortem and PET studies (113) indicates that depression is associated with elevated proinflammatory markers in the brain. However, studies have not yet established a correlation between the levels of peripheral and central inflammatory markers in depressed patients (110). The development of novel PET radioligands that specifically bind to markers of activated microglia and astrocytes offers a promising solution for quantifying neuroinflammation *in vivo* (114). Recent study findings indicate that elevated brain TSPO binding (a putative marker of microglia-related inflammatory processes) in MDD patients, may serve as an indicator of susceptibility to heightened suicidal ideation and negative affect following acute stress (115). Furthermore, Eiff and colleagues demonstrated a promising TSPO-PET imaging approach to bridge the gap between systemic peripheral inflammation and neuroinflammation in MDD patients, highlighting the skull bone marrow and venous sinuses as critical sites for peripheral-central immune communication (116). Depression associated with inflammation may be perceived as an inflammatory condition where systemic and central inflammation converge via multiple pathways to disrupt neural function, although definitive evidence linking peripheral markers to central processes in living patients remains a significant technical challenge. Clinical evidence indicates that elevated levels of inflammatory mediators are both associated with and can induce depressive symptoms. This supports the etiological validity of experimental models that utilize inflammatory stimuli to recapitulate depressive-like behavior. This data provides the basis for animal models of depression that involve administering inflammatory inducers or proinflammatory cytokines. A key unresolved question, however, is whether the molecular pathways activated in these models, including central nervous system events, accurately recapitulate those underlying the development of depression in patients.

### 2.8 LPS

The most widely employed inducers of inflammation in depression models are bacterial LPSs, typically derived from Escherichia coli. Peripheral LPS administration may also model conditions of increased intestinal permeability, a hypothesized contributor to depression. Lipopolysaccharides, also known as endotoxins, are components of the outer membrane of gram-negative bacteria. They are potent systemic immune activators capable of inducing pro-inflammatory states ranging from fever to septic shock (10). LPS effects extend to neurotransmission, where it alters monoamine levels, decreasing dopamine and serotonin while increasing norepinephrine in key brain regions (117). Although LPS activates the immune system across all mammals, sensitivity and transcriptional responses vary significantly by species. For instance, gene expression patterns during endotoxemia show weak correlation between mice and humans (118). Nevertheless, LPS administration remains the predominant experimental model for inducing a depressive-like state in both rodents and humans. This preeminence is attributable to several distinct methodological and practical advantages.

A key advantage of the LPS model is its high degree of standardization. Commercially available LPS, derived from various bacterial strains, enables consistent protocols across laboratories. Following intravenous or intraperitoneal administration, circulating endotoxin levels can be quantified reliably using assays such as the LAL test or ELISA (119). The model is also characterized by a favorable safety profile. Low LPS doses are well tolerated in both humans and animals. Notably, endotoxin is detectable at low concentrations in healthy individuals. Although values show considerable inter-individual variability, 98% of donors have levels below 1 EU/ml, with a reported average plasma concentration of 0.128 EU/ml (120). According to the WHO standard, 1 EU is equivalent to 120 pg of E. coli O113:H10:K LPS (121). Thus, the average healthy level corresponds to approximately 15.36 pg/ml. This baseline is markedly lower than levels observed in pathological states. For instance, the average plasma endotoxin concentration in patients with sepsis is 581 pg/ml (122), which is nearly 40 times higher than the healthy average.

Current evidence indicates that LPS at low to moderate doses does not cross the BBB in substantial amounts. For instance, only approximately 0.025% of intravenously administered labeled LPS penetrates the BBB in mice (123). The limited LPS that does enter the brain likely does so via specific transport mechanisms, such as lipoprotein-mediated transport. Within the brain, peripherally administered LPS is found primarily in astrocytes, tanycytes, and the endothelial cells of circumventricular organs (124). In contrast, high LPS doses compromise BBB integrity. In mice, a dose of 3 mg/kg increases BBB permeability to both low-molecular-weight sucrose and high-molecular-weight albumin, whereas lower doses (0.3 and 0.03 mg/kg) do not produce this effect (125). LPS is the main experimental tool for modeling inflammation-associated depression. Its utility stems from a potent, standardized, and dose-dependent ability to induce systemic immune response that culminates in a depressive-like behavior, even though its direct brain penetration is minimal at relevant doses.

## 3 Inflammatory models in humans

### 3.1 Experimental endotoxemia in humans

#### 3.1.1 Effects of LPS

Low-dose LPS administration in humans is safe and well-tolerated. It reliably induces a transient systemic inflammatory response characterized by elevated blood proinflammatory cytokines and flu-like sickness symptoms. A subset of these induced symptoms, including low mood, fatigue, changes in appetite, and cognitive impairment, closely overlaps with some symptoms of clinical depression. However, endotoxemia does not replicate feelings of worthlessness, hopelessness, guilt, or suicidal ideation (126). This discrepancy likely reflects the profound difference in duration between an acute experimental challenge and the chronic course of clinical depression. It is assumed that inflammation in humans is associated exclusively with specific symptoms, the majority of which are related to sickness behavior (127). This significant, albeit incomplete, symptomatic overlap provides a strong rationale for using experimental endotoxemia to model aspects of a depressive-like state in humans. This symptomatic overlap provides a valuable platform for developing and testing therapeutics targeted at inflammation-associated depression (128).

A substantial body of research has investigated the effects of endotoxin administration in humans (see **Table 2**). Collectively, they show highly consistent results, with all measured parameters, cytokine and hormone concentrations and symptom severity, exhibiting clear dose dependency. Higher LPS doses lead to greater increases in cytokine levels (129), and more pronounced activation of the HPAA, as measured by ACTH and cortisol (130). The inflammatory and neuroendocrine responses follow a characteristic temporal profile. Blood TNF-α peaks at 1–2 hours post-administration, followed by IL-6 and IL-10 at 2–3 hours. Norepinephrine levels rise by 2 hours, with cortisol peaking later, at 3–4 hours. Notably, the cytokine levels induced in experimental endotoxemia are substantially higher than those typically observed in patients with depression (see **Table 3**). This suggests that the model elicits a more potent immune activation than is generally present in the clinical state of depression.

**Table 2:** Experimental endotoxemia in humans.

Participants	LPS dose	Serotype	Injection site	Experimental design	Analysis time after injection	Methods for assessing behavioral changes	Behavioral outcomes	Blood analytes	References
male and female	2.0 ng/kg	Escherichia coli, Cat №: 1235503, lot H0K354, United States Pharmacopeia	IV	acute LPS treatment	0-7 h following the injection	SicknessQ	↑Sickness symptoms	↓tryptophan, ↓kynurenine, ↓nicotinamide, ↑quinolinic acid, - kynurenic acid, - 3- hydroxykynurenine, -picolinic acid	(342)
male	0.8 ng/kg	Escherichia coli (O113:H10)	IV	acute LPS treatment	30 min before the injection and 1-72 h following the injection	GASE, Multidimensional Mood Questionnaire, SSS, STADI, STAI	↑Sickness symptoms, ↓alertness, ↓calmness, ↓positive mood, ↑negative mood, ↑sleepiness, ↑state anxiety	↑IL-6, ↑IL-1RA, ↑CRP, ↑cortisol	(148)
male and female	0.8 ng/kg	Escherichia coli group O:113: BB-IND 12948 to MRI	IV	acute LPS treatment	2 h following the injection	Monetary reward task in the fMRI scanner	↓Ventral striatum activity in anticipation of reward	↑IL-6, ↑TNF-α	(157)
male and female	0.8 ng/kg	Escherichia coli group O:113: BB-IND 12948 to MRI	IV	acute LPS treatment	baseline and 1-6 h following the injection	Depression subscale of the short-form POMS	↑Depressed mood	↑IL-6, ↑TNF-α	(145)
male	2 ng/kg	Escherichia coli O113	IV	acute LPS treatment	baseline and 15, 30, 45, 60, 90, 120, 180, 240, 360 min, 24 h, 48 h following the injection	not assessed	–	↑IL-6, ↑CRP, ↓tryptophan, ↑kynurenine, - kynurenic acid, - 3-hydroxykynurenine, ↑quinolinic acid, - picolinic acid	(343)
male and female	0.8 ng/kg	Escherichia coli O113	IV	acute LPS treatment	baseline and 2 h, 6 h following the injection	Depression subscale of the short-form POMS	↑Depressed mood	↑IL-6, ↑TNF-α, ↓tryptophan, ↑kynurenine, ↑ kynurenic acid, - quinolinic acid	(344)
male and female	0.8 ng/kg	Escherichia coli, Cat №: 1235503, lot H0K354, United States Pharmacopeia	IV	acute LPS treatment	30 min before the injection and 1-6 h and 24 h following the injection	SicknessQ, MDBF, STAI, VAS of Fatigue	↑Sickness symptoms, ↑fatigue, ↑negative mood, ↑state anxiety	↑IL-6, ↑TNF-α, ↑IL-10, ↑CRP, ↑cortisol, ↑norepinephrine	(142)
male and female	0.8 ng/kg	Escherichia coli group O:113	IV	acute LPS treatment	baseline and 1-6 h following the injection	SicknessQ, POMS, Cyberball social exclusion task in the fMRI scanner	↑Depressed mood	↑IL-6, ↑cortisol	(131)
male and female	0.6 ng/kg	Escherichia coli, lot G3E069, United States Pharmacopeia	IV	acute LPS treatment	baseline and 1.5 h, 3.5 h, 5 h following the injection	SicknessQ, STAI, MRI	↑Sickness symptoms, ↑state anxiety	↑TNF-α, ↑IL-6	(144)
male and female	0.8 ng/kg	NIH Clinical Center Reference LPS	IV	acute LPS treatment	baseline and 60, 90, 120, 180, 240 min following the injection	MADRS, POMS, VAS	↑Lassitude, ↑anhedonia, ↑fatigue, ↓vigor, ↓social interest	↑IL-1β, ↑IL-2, ↑IL-6, ↑IL-10, ↑IL-12 p70, ↑ IFN-γ, ↑TNF-α, ↑GM-CSF, ↑CXCL8, ↑CXCL10, ↑CCL2, ↑CCL4, ↑CCL11, ↑CCL13, ↑CCL17	(134)
male	1 and 2 ng/kg	US Standard Escherichia coli O:113	IV	acute LPS treatment and an initial endotoxin bolus of 1 ng/kg followed by 1 ng/kg/h for 3 hours (cumulative dose of 4 ng/kg)	baseline and 1-8 h following the injection	flu-like symptoms assessment	↑Flu-like symptoms	↑TNF-α, ↑IL-6, ↑CXCL8, ↑IL-10, ↑IL-1β, ↑MIP-1α, ↑MIP-1β, ↑MCP-1	(163)
male and female	0.8 ng/kg	NIH Clinical Center Reference LPS	IV	acute LPS treatment	baseline and 1 h, 2 h, 3 h following the injection	MADRS, STAI, VAS	No effect on anxiety, ↑lassitude, ↑social anhedonia	↑TNF-α, ↑IL-6	(317)
male	0.4 ng/kg	Escherichia coli, lot G3E0609, United States Pharmacopeia	IV	acute LPS treatment	0.25 h before and 1, 1.75, 3, 4, 6, 24 h following the injection	MDBF, STAI, Social cognition task in the fMRI scanner	↑State anxiety, ↓alertness, ↓calmness, ↓positive mood	↑IL-6, ↑TNF-α, ↑IL-10, ↑IL-1RA	(136)
male and female	0.6 and 0.8 ng/kg	Escherichia coli, United States Pharmacopeia	IV	acute LPS treatment	baseline and 1-6 h following the injection	Likert scale, KSS, SSS	↑Fatigue, ↑sleepiness	↑TNF-α, ↑IL-6	(143)
male and female	0.8 ng/kg	NIH Clinical Center Reference LPS	IV	acute LPS treatment	baseline and 60, 90, 120, 180 min following the injection	MADRS, POMS, VAS, scales to rate headache, muscle aches, chills, nausea, ^18^F-FDG PET	↑Fatigue, ↓social interest, ↑lassitude	↑TNF-α, ↑IL-6, ↑cortisol	(133)
male and female	0.8 ng/kg	Escherichia coli group O:113	IV	acute LPS treatment	baseline and 1-6 h following the injection	Likert scale, POMS, Self-reported sickness symptoms assessment	↑Sickness symptoms, ↑social disconnection, ↑depressed mood	↑TNF-α, ↑IL-6	(152)
male and female	0.8 ng/kg	Escherichia coli group O:113	IV	acute LPS treatment	baseline and 1-6 h following the injection	Self-Reported Sickness Symptoms assessment, POMS, monetary reward task in the fMRI scanner	↑Sickness symptoms, ↑depressed mood	↑TNF-α, ↑IL-6	(156)
male and female	0.4 ng/kg	Escherichia coli, lot H0K354, United States Pharmacopeia	IV	acute LPS treatment	baseline and 1-6 h following the injection	STAI, MDBF, GASE	↑State anxiety, ↑sickness symptoms, ↑fatigue, ↓positive mood, ↓calmness	↑TNF-α, ↑IL-6, ↑IL-10, ↑IL-1RA, ↑dehydroepiandrosterone, ↑noradrenaline, ↑cortisol, ↑prolactin	(139)
male and female	0.4, 0.6 and 0.8 ng/kg	Escherichia coli, United States Pharmacopeia	IV	acute LPS treatment	baseline and 2 h, 3 h, 6 h following the injection	STAI	↑State anxiety	↑TNF-α, ↑IL-6, ↑IL-10	(140)
male	0.4 ng/kg	Escherichia coli, lot G3E069, United States Pharmacopeia	IV	acute LPS treatment	baseline and 1-6 h following the injection	Visual stimulation and an emotion processing task in the fMRI scanner, STAI, MDBF	↑State anxiety, ↓positive mood, ↓calmness, ↓alertness	↑TNF-α, ↑IL-1RA, ↑IL-6, ↑IL-10, ↑cortisol	(135)
male	0.8 ng/kg	Escherichia coli, lot H0K354, United States Pharmacopeia	IV	acute LPS treatment	baseline and 1 h, 2 h, 3 h, 4 h, 6 h, 24 h following the injection	STADI	↑Dysthymia	↑TNF-α, ↑IL-6, ↑IL-10, -IL-1β, ↑CRP, -albumin, -IgG	(159)
male and female	0.8 ng/kg	Escherichia coli group O:113: BB-IND 12948 to MRI	IV	acute LPS treatment	baseline and 1 h, 2 h, 3 h, 4 h, 6 h following the injection	Self-Reported Sickness Symptoms assessment, POMS, Questionnaire for social disconnection assessment	↑Depressed mood, ↑feelings of social disconnection, ↑sickness symptoms	↑TNF-α, ↑IL-6	(153)
male and female	0.8 ng/kg	Escherichia coli group O:113: BB-IND 12948 to MRI	IV	acute LPS treatment	baseline and 1-6 h following the injection	POMS, Physical sickness symptoms assessment	↑Sickness symptoms, ↑depressed mood	↑TNF-α, ↑IL-6	(150)
male and female	0.8 ng/kg	Escherichia coli group O:113	IV	acute LPS treatment	2 h following the injection	Motivation to approach support figure assessment, viewing support figures and strangers in the fMRI scanner	↑Self-reported desire to be around the support figure	↑TNF-α, ↑IL-6	(155)
male	0.8 ng/kg	Salmonella abortus equi endotoxin	IV	acute LPS treatment	baseline and 1 h, 3 h, 9 h following the injection	Depression Adjectives Check List, State Anxiety Inventory, Story Recall test, Figure Recall test, Word List Learning test, Ruff 2 and 7 cancellation test, Digit Span Forward test, Digit Symbol test, Simple Reaction time test, Continuous Performance test, TMT A and B test, Word Fluency test, Physical sickness symptoms assessment	↑Depressed mood, ↑state anxiety, no effects on physical sickness, attention and executive functions, ↓memory functions	↑TNF-α, ↑sTNF-R p55, ↑sTNF-R p75, ↑IL-1RA, ↑IL-6, ↑cortisol	(345)
male	0.4 and 0.8 ng/kg	Escherichia coli, lot G3E069, United States Pharmacopeia	IV	acute LPS treatment	baseline and 1, 1.75, 3, 4, 6, 24 h following the injection	STAI, MDBF, N-back task, Recall of emotional and neutral pictures of the International Affective Picture System	↑State anxiety, ↓alertness, ↓calmness, ↓positive mood	↑TNF-α, ↑IL-6, ↑IL-10, ↑IL-1RA, ↑cortisol, ↑norepinephrine	(132)
male	0.8 ng/kg	Escherichia coli, serotype O113:H10:K-negative, lot H0K354, United States Pharmacopeia	IV	acute LPS treatment	baseline and 1, 2, 3, 4, 6 h following the injection	STADI, GASE, affective Go/No-Go task	↑Sickness symptoms, ↓euthymia, ↑dysthymia	↑TNF-α, ↑IL-6	(141)
male	2 ng/kg	Escherichia coli (not specified)	IV	acute LPS treatment	baseline and 30 min-8 h following the injection	Effort-stake task, POMS, Physical sickness symptoms assessment	↑Sickness symptoms, ↑self-reported depression and fatigue	↑TNF-α, ↑IL-6	(147)
male and female	0.8 ng/kg	not specified	IV	acute LPS treatment	baseline and 1-6 h following the injection	Social Feedback fMRI Task	↑Sensitivity to social information	↑TNF-α, ↑IL-6	(154)
male	1.0 ng/kg	NIH Clinical Center Reference LPS Escherichia coli group O:113	IV	acute LPS treatment	baseline and 60, 90, 120, 180, 240 min following the injection	Physical sickness symptoms assessment	↑Sickness symptoms	↑TNF-α, ↑IL-6, ↑IL-8, ↑IL-10, - IFN-γ	(158)
male and female	2 ng/kg	Escherichia coli, lot H0K354, United States Pharmacopeia	IV	acute LPS treatment	baseline and 1-7 h following the injection	SicknessQ, KSS, STAI, Swedish Core Affect Scale (SCAS), Effort Expenditure for Rewards Task (EEfRT)	↑Sickness symptoms, ↑state anxiety, ↑sleepiness, ↓vigor, ↓positive affect, ↑behavioral performance in high effort/high reward trials	↑IL-6	(146)
male and female	0.4 and 0.8 ng/kg	Escherichia coli, serotype O113:H10:K-negative, United States Pharmacopeia	IV	acute LPS treatment	baseline, 1-6 h and 24 h following the injection	GASE, STAI, MDBF	↑State anxiety, ↓positive mood, ↓alertness, ↓calmness, ↑sickness symptoms	↑TNF-α, ↑IL-6, ↑CRP, ↑cortisol	(149)
male	0.4 and 0.8 ng/kg	Escherichia coli, lot G3E069, United States Pharmacopeia	IV	acute LPS treatment	baseline and 1-6 h following the injection	STAI, MDBF, pain assessment	↓Positive mood, ↓calmness, ↓alertness, ↑state anxiety, ↑pain sensitivity	↑TNF-α, ↑IL-6, ↑IL-8, ↑IL-10, ↑cortisol	(137)
male	0.4 ng/kg	Escherichia coli, lot G3E069, United States Pharmacopeia	IV	acute LPS treatment	baseline and 1-6 h following the injection	MDBF, visceral and somatic pain assessments	↓Positive mood, ↓alertness, ↑pain sensitivity	↑TNF-α, ↑IL-6, ↑cortisol	(138)

POMS, Profile of Mood States; STAI, State-Trait Anxiety Inventory; STADI, State Trait Anxiety Depression Inventory; SicknessQ, Sickness Questionnaire; MADRS, Montgomery-Åsberg Depression Rating Scale; MDBF, German multidimensional mood questionnaire/German version of the POMS; GASE, General-Assessment-of-Side-Effects questionnaire; VAS, Visual Analog Scale; SSS, Stanford Sleepiness Scale; KSS, Karolinska Sleepiness Scale.

↑ increase; ↓ decrease; – no change.

**Table 3:** Cytokine levels during human endotoxemia.

Participants	LPS dose	Methods and kits for cytokine measurement	Sample type	IL-6, pg/ml	TNF-α, pg/ml	IL-1RA, pg/ml	IL-10, pg/ml	References
healthy subjects	placebo	ELISA (R&D Systems)	serum	1.3 ± 0.4	2 ± 0.6	not determined	not determined	(317)
	0.8 ng/kg			197.9 ± 47.9*	45.2 ± 10.8*	not determined	not determined	
healthy subjects	placebo	Bio-Plex Cytokine Assays, Bio-Rad Laboratories	plasma	2.71	20.06	0.07 ng/ml	0.90	(136)
	0.4 ng/kg			144.25*	71.12*	5.59 ng/ml*	23.94*	

*Significant differences, P value < 0.05.

Sickness symptoms emerge 2–3 hours following LPS administration, coinciding with the peak experience of fatigue. Mood deterioration and increased anxiety manifest 2–3 hours after administration (131–144). Furthermore, endotoxin administration induces a transient depressive mood state (145) and impairs motivation (146, 147). Most of these behavioral and biochemical parameters return to baseline within six hours. A notable exception is CRP, which peaks in concentration approximately 24 hours post-administration (148). While some residual sickness symptoms may persist at 24 hours, their intensity is markedly diminished (149).

The study results indicate that a depressive mood state develops in 36% of individuals following LPS administration (150). LPS administration selectively reduces food consumption without altering water intake, an effect associated with endotoxin-induced secretion of TNF-α and IL-6 (151). Furthermore, the inflammatory challenge increases feelings of social disconnection (loneliness) within two hours (152, 153). Neuroimaging studies demonstrate that endotoxin administration in humans alters functional activity in key brain regions (dACC, amygdala, ventral striatum, and VMPFC), which are central to processing pain, threat, and reward. Following this inflammatory challenge, these regions exhibit heightened sensitivity to both positive and negative social feedback (154). Endotoxin potentiates ventral striatal activity in response to images of support figures and increases self-reported desire for social support (155).

Conversely, the response to nonsocial reward is blunted. Ventral striatal activity decreases in response to monetary reward cues in subjects exposed to endotoxin (156, 157).

Evidence indicates that peripheral LPS administration induces neuroinflammation in humans. PET studies show that LPS significantly increases microglial activation within three hours of administration. This central immune response coincides with elevated blood levels of inflammatory cytokines (TNF-α, IL-6, IL-8, and IL-10) and the onset of sickness symptoms (158). Notably, IL-6 levels in the CSF peak six hours post-administration, a time point when peripheral IL-6 has returned to baseline. A strong positive correlation exists between the magnitude of the CSF IL-6 increase and the severity of mood impairment (159).

Experimental endotoxemia with low-dose LPS is a reproducible, standardized, and ethically justifiable model for studying sickness and depressive-like states in humans. The symptoms induced by LPS administration show a significant but incomplete overlap with those of clinical depression, providing a crucial translational bridge for investigating immune-mediated depressive states.

#### 3.1.2 Sex differences in LPS effects in humans

It is known that depression is more common in women than in men. This observed gender gap may be substantially attributable to the under-recognition of depression in men, stemming from diagnostic biases, gender-specific symptom presentation, and help-seeking strategies. Some researchers suggest that depression in men frequently presents with non-canonical symptom profile, which may contribute to its underdiagnosis. This often includes chronic anger, self-destructiveness, drug use, gambling, womanizing, and workaholism. These manifestations are poorly captured by standard diagnostic criteria for Major Depressive Disorder. This discrepancy highlights a potential gender bias in current diagnostic frameworks and a “masked” clinical presentation in male populations (160). Women are generally more likely to recognize mild-moderate depression than men. This can be explained by different coping styles. Male subjects intend to “omit” symptoms, while females “notice” symptoms. Women are more willing to express affective symptoms and seek medical help. Their symptomatic profile often aligns more closely with current diagnostic criteria for Major Depressive Disorder and frequently includes somatic complaints (e.g., fatigue, sleep disturbance). Conversely, men exhibit higher levels of mental health stigma being emotionally unexpressive and reluctant to seek help. Men exhibit more atypical symptoms like aggression or substance addiction which are poorly captured by standard diagnostic criteria. This contributes to the systematic underdiagnosis and misdiagnosis of depression in male populations (161). Some research indicates that females experience a greater increase in depressed mood and feelings of social disconnection following LPS administration, despite showing no difference in peripheral cytokine levels compared to males (153). Conversely, other work finds no sex differences in LPS-induced mood or anxiety changes but reports a more robust inflammatory and neuroendocrine response in females, characterized by significantly greater elevations in blood TNF-α, IL-6, and cortisol (139). Human experimental endotoxin challenge studies typically gender-balanced. However, the behavioral outcome often relies on subjective self-report via verbal interview. This methodology may be systematically biased by gender differences in symptom reporting and expressiveness. Specifically, men may be more likely to minimize or underreport depressive symptoms. In this context, objective instrumental methods, which are not contingent upon subjective mood states, provide a more reliable and valid measure of the physiological state.

#### 3.1.3 Methods of LPS administration and doses

The majority of human experimental endotoxemia studies employ a bolus intravenous injection of LPS. To prolong the inflammatory response, some protocols utilize continuous intravenous infusion. Bolus injection elicits a more pronounced and dynamic cytokine response than a four-hour continuous infusion. Specifically, peak plasma levels of TNF-α occur at 2 hours post-bolus versus 5 hours post-infusion, while IL-6 peaks at 3 and 6 hours, respectively (162). Combining a bolus with a continuous infusion yields an even greater increase in plasma cytokines and prolongs flu-like symptoms (163).

The most frequently administered LPS dose range is 0.4–0.8 ng/kg. Doses of 1–4 ng/kg, while sometimes used, are associated with a higher incidence of adverse effects, including headache, arthralgia, fever, dizziness, and, less commonly, chills, nausea, and vomiting (164).

Human experimental endotoxemia studies are characterized by a high degree of methodological standardization. Protocols typically involve a narrow intravenous dose range (0.4–2 ng/kg) of Escherichia coli O:113 LPS and include both male and female participants. Assessments are generally confined to a 6-hour post-administration window, using standardized scales to quantify sickness symptoms and depressed mood. This rigorous standardization enables reliable comparisons across studies conducted by different research groups and contributes to the high reproducibility of results.

A significant limitation of this model is its acute nature, which contrasts with the chronic and recurrent course of clinical depression, which persists for weeks to years. In principle, chronic LPS administration could address this disparity, but this approach is precluded by the rapid development of immunological tolerance upon repeated LPS administration (165).

LPS-induced effects are notably transient, usually resolving completely within six hours. This short time frame precludes the evaluation of treatments that require chronic administration. Furthermore, administering antidepressants before LPS exposure, a common preclinical design, does not replicate the clinical sequence where treatment follows symptom onset. While the model reliably reproduces sickness behavior and one core symptom of depression (i.e., low mood), the induced mood disturbance is short-lived and coincides temporally with sickness symptoms. Experimental endotoxemia fails to capture most other diagnostic symptoms of depression, which either do not manifest, are not assessed, or cannot be adequately evaluated within this brief window. Notably, anhedonia is not robustly reproduced, although some studies report alterations in reward sensitivity (166). A significant methodological constraint in human studies is the reliance on peripheral blood for molecular and biochemical analyses, as invasive sampling of brain tissue is not feasible. PET studies confirm that LPS administration activates brain immune cells (158, 167), suggesting a concurrent increase in inflammatory mediators within the brain. Furthermore, depressive symptoms in humans are assessed via self-report scales and questionnaires rather than objective instrumental measures. This reliance on subjective metrics restricts the ability to elucidate the underlying pathophysiological mechanisms of depression using the human experimental endotoxemia model. To circumvent this limitation, future research should prioritize the advancement of non-invasive neuroimaging technologies and the development of novel, cell-specific radioligands. Specifically, next-generation PET tracers with higher specificity and affinity for activated microglia and astrocytes are critically needed. A parallel and critical research area is the discovery and validation of novel highly specific biomarkers in cerebrospinal fluid and blood that more accurately reflect the pathophysiological state of the central nervous system during depressive episodes.

Nevertheless, this model may provide valuable insight into the molecular mechanisms underlying the development of the inflammatory subtype of depression. For example, analysis of the blood cell transcriptome before LPS administration revealed a link between depressed mood after LPS administration and increased activity of inflammation-related transcription factors (nuclear factor kappa B, NF-kB) and beta-adrenergic signaling (cAMP response element binding protein, CREB), as well as decreased activity of glucocorticoid receptors (a marker of glucocorticoid resistance) (150).

#### 3.1.4 Sensitivity to endotoxin in humans

In human experimental studies, administered LPS doses typically range from 0.4 to 2 ng/kg, with 0.8 ng/kg being the most frequently employed dose. The lethal LPS dose for humans is not established. The effects of high doses can only be inferred from rare, uncontrolled reports. A 2 µg intravenous dose caused hypotension, fever, chills, and required intensive care. The person’s weight is not indicated in the report, and therefore it is impossible to calculate the dose, but if we take the average weight of a man equal to 85 kg, then the administered dose is more than 20 ng/kg (168). Another well-known example is the endotoxin administration to humans for therapeutic purposes. A laboratory worker had administered 1 mg of Salmonella Minnesota endotoxin intravenously in an attempt to treat a recently diagnosed tumor. The person was admitted to the intensive care unit with signs of septic shock with a temperature of 40^0^C and a pulse of 114 per minute. The amount of endotoxin administered is equivalent to a dose of 15,000 ng/kg, which is 3,750 times greater than the dose of 4 ng/kg, which is also used in research (169). Controlled studies confirm the dose-dependent nature of effects, with the minimum pyrogenic dose in humans falling between 0.1 and 0.5 ng/kg (170). The reviewed data establish low-dose LPS administration as a safe and controlled experimental model in humans. However, they simultaneously delineate a critical safety threshold, demonstrating that extremely high doses can precipitate life-threatening states.

### 3.2 Typhoid vaccination

A strategic alternative to overcome the acute regimen of LPS model and transient nature of LPS-induced effects is the use of alternative immune stimuli (e.g., typhoid vaccine, live attenuated vaccines). Vaccination induces a subacute, milder, and more prolonged immune activation that better mimics the low-grade, sustained inflammation observed in a subset of patients with depression. For instance, typhoid vaccination provides a human experimental model for inducing a depressive-like state, one that is considered milder than the LPS model. Intramuscular administration increases IL-6 levels and induces negative mood changes 3 hours post-injection (171, 172). Vaccine-induced inflammation heightens sensitivity to aversive stimuli while reducing sensitivity to rewarding stimuli (173). Unlike the LPS model, it does not increase physical symptoms or body temperature (174). This model is rarely used, and its mood-altering effects are not consistently reproducible. Some studies report increased IL-6 levels without corresponding mood changes (175, 176). All studies employ the same vaccine type and standard intramuscular dose, with assessment periods not exceeding 8 hours (see **Table 4**). Consequently, this model offers no advantage for modeling prolonged, low-grade inflammation or a sustained depressive-like state. The typhoid vaccination model induces a mild, transient inflammatory state in humans that can alter mood and reward processing, but its effects are inconsistent and less robust than those of the LPS model.

**Table 4:** Typhoid vaccination.

Participants	Vaccine dose	Vaccine	Injection site	Experimental design	Analysis time after injection	Methods for assessing behavioral changes	Behavioral outcomes	Blood analytes	References
male	0.025 mg	Salmonella typhi capsular polysaccharide vaccine (Typhim Vi, Aventis Pasteur MSD, Berkshire, United Kingdom)	IM	acute treatment	baseline and 2 h, 3 h following the injection	POMS, face perception task in the fMRI scanner	↑Mood deterioration	- TNF-α, ↑IL-6, - IL-1RA, - cortisol	(172)
male	0.025 mg	Salmonella typhi capsular polysaccharide vaccine (Typhim Vi, Sanofi Pasteur, UK)	IM	acute treatment	baseline and 5.5 h, 8 h following the injection	POMS, Reading the Mind in the Eyes test	No effects on sickness symptoms and mood	↑IL-6	(176)
male and female	0.025 mg	Salmonella typhi capsular polysaccharide vaccine (Typhim Vi, Aventis Pasteur MSD)	IM	acute treatment	baseline and 1-8 h following the injection	POMS	No effects on sickness symptoms, ↑negative mood	not determined	(174)
male and female	0.025 mg	Salmonella typhi capsular polysaccharide vaccine	IM	acute treatment	2.5 – 3.5 h following the injection	POMS, Reinforcement Learning Task in the fMRI scanner	No effects on sickness symptoms, ↑fatigue, shift in reward versus punishment sensitivity	↑IL-6	(173)
male	0.025 mg	Salmonella typhi capsular polysaccharide vaccine (Typhim Vi)	IM	acute treatment	baseline and 1.5 h, 3 h, 6 h following the injection	POMS	↑Negative mood	- TNF-α, ↑IL-6, - IL-1RA	(171)
male	0.025 mg	Salmonella typhi capsular polysaccharide vaccine (Typhim Vi, Aventis Pasteur MSD)	IM	acute treatment	baseline and 30, 60, 90, 150 min following the injection	POMS	↑Negative mood	↑IL-6	(175)

IM, Intramuscular; POMS, Profile of Mood States.

↑ increase; – no change.

## 4 Inflammatory models in rodents

As in humans, various immune system stimulants are used in animals to study the role of inflammation in the development of a depressive-like state. Several inflammatory models of depression exist in rodents. The most common is the LPS-induced model, based on bacterial endotoxin administration (see **Tables 5**, **6**). Other models include cytokine-induced models (based on the TNF-α, IL-1β, or IL-6 administration), an IFN-α-induced model, and a tuberculosis vaccine-induced model. Compared to the CUMS paradigm, acute LPS administration in mice typically produces more severe depressive-like behavior and a more pronounced increase in cytokine levels in both the brain and periphery (177).

**Table 5:** Experimental endotoxemia in mice.

Animals	Sex	LPS dose	Serotype	Injection site	Experimental design	Analysis time after injection	Behavioral tests	Behavioral outcomes	References
C57BL/6J mice	male	0.5 mg/kg	Escherichia coli (O55:B5)	IP	chronic (7 days) and acute LPS treatment	24 h following the last injection	SPT, FST	↓sucrose preference, ↑immobility time in the FST	(16)
ICR mice	male	0.83 mg/kg	Escherichia coli (0111:B4)	IP	acute LPS treatment	24 h following the injection	SPT, FST, OFT, TST	↑immobility time in the FST and TST, ↓sucrose preference, no effect in the number of crossing and the number of rearing in the OFT	(346)
C57BL/6 mice	male	0.83 mg/kg	Escherichia coli (O55:B5)	IP	acute LPS treatment	24 h following the injection	SPT, FST, OFT, TST, EPM	↓time spent in the center area of the OFT and entries to the center area of the OFT, ↓time spent in the open arms of the EPM and entries to the open arms of the EPM, ↓sucrose consumption, ↑immobility time in the FST and TST	(347)
C57BL/6 mice	female	2 mg/kg	Escherichia coli (O55:B5)	IP	chronic (5 days) LPS treatment	10-11 days following the last injection	OFT, EPM, FST, TST	no effect in the total distance traveled in the OFT, ↓time in the central area of the OFT, ↑immobility time in the FST and TST, ↓time spent in the open arms of the EPM	(348)
C57BL/6 mice	male and female	0.83 mg/kg	Escherichia coli (O127:B8)	IP	acute LPS treatment	24 h following the injection	OFT, FST	↑immobility time in the FST (only in males), ↓locomotor activity in the OFT (only in females), ↓total distance traveled in the OFT (only in females), no effect in the number of entries into the center square of the OFT	(349)
Swiss mice	female	schedule of doses was the following: day 1 – 0.75 mg/kg, day 2 – 1 mg/kg, day 3 – 1.25 mg/kg, day 4 – 1 mg/kg, day 5 – 0.75 mg/kg	Escherichia coli (O55:B5)	IP	chronic LPS treatment (injection of LPS were given for five consecutive days during the first week of each month and repeated for four months)	24 h and 6 weeks following the last injection	SPT, FST, OFT	↑immobility time in the FST, ↓sucrose preference, no effect in the number of crossings in the OFT	(201)
ICR mice	male	low-dose LPS (0.2 mg/kg), high-dose LPS 8.3 mg/kg	Escherichia coli (O127:B8)	IP	high-dose LPS in a 6 days after preconditioning with low-dose LPS	24 h following the last injection	FST, OFT	↑immobility time in the FST, ↓distance traveled in the OFT	(222)
C57BL/6J mice	male	0.5 mg/kg	Escherichia coli (O55:B5)	IP	acute LPS treatment	24 h following the injection	FST, OFT, TST	↑immobility time in the FST, ↑immobility time in the TST, ↑time in the periphery in OFT, ↓time in the center in OFT	(177)
C57BL6/J mice and *Ido1*^−/−^ mice	male and female	0.33, 0.5, 1 mg/kg	Escherichia coli (O127:B8)	IP	acute LPS treatment	24 h following the injection	FST, SPT, LMA, NSFT, anticipatory activity task, progressive ratio schedule of food reinforcement, effort-based decision making task, CPP	↓locomotor activity in the OFT, ↑immobility time in the FST, ↓sucrose preference, ↑latency to feed in the NSF task, ↓total effort in a progressive ratio task, ↓total nose pokes in effort-based decision making task	(350)
NMRI mice	male	0.83 mg/kg	Escherichia coli (O55:B5)	IP	acute LPS treatment	24 h following the injection	SPT, TST	↓sucrose preference, ↑immobility time in the TST	(180)
BALB/c mice	male	0.5 mg/kg	Escherichia coli (O127:B8)	IP	acute LPS treatment	24 h following the injection	TST, LMA	no effects on spontaneous locomotor activity, ↑immobility time in the TST	(319)
C57BL/6J mice	female	0.1 mg/kg	Escherichia coli (O127:B8)	IP	chronic (4 days) LPS treatment	24 h after each LPS dose for SPT and 22 h after the third LPS dose for FST	SPT, FST	↓sucrose preference, ↑immobility time in the FST, ↓climbing time in the FST	(322)
C57BL/6 mice	male and female	day 1 – 0.75 mg/kg, day 2 – 1 mg/kg, day 3 – 1.25 mg/kg, day 4 – 1 mg/kg, day 5 – 0.75 mg/kg	Escherichia coli (O127:B8)	IP	repeated LPS injections once daily for 5 days at a one-month interval repeated for 4 consecutive months	not specified	SPT	↓sucrose preference (only in females)	(202)
C57BL/6J mice	male	0.052, 0.104, 0.208, 0.415, 0.83 mg/kg	Escherichia coli (O55:B5)	IP	chronic (3-5 days) and acute LPS treatment	6 h, 24 h, 48 h following the last injection	SPT, FST, OFT, rotarod, female urine sniffing test	↓distance traveled in the OFT, no effects on immobility time in the FST, ↓sucrose preference, ↓time spent sniffing urine	(214)
Swiss mice	male	0.1 mg/kg	Escherichia coli (O127:B8)	IP	chronic (every other day, 8 times) LPS treatment	24 h following the injection	FST, OFT, TST, MWM	↑immobility time in the FST, ↑immobility time in the TST, ↓number of rearings in the OFT, no effects in numbers of crossings in the OFT, ↓quadrant dwell time in the MWM, ↓numbers of platform crossings in the MWM	(198)
Swiss mice	male	0.1 mg/kg	Escherichia coli (O26:B6)	IP	acute LPS treatment	2 h following the injection	FST, OFT, TST, LDB	↑immobility time in the FST, ↑immobility time in the TST, ↓number of line crossings in the center and in the periphery in the OFT, ↓total number of line crossings and number of rears in the OFT, ↓number of transitions between the light and dark compartments in the LDB	(351)
C57BL/6 and CD1 mice	female	0.83 mg/kg	Escherichia coli (O127:B8)	IP	acute LPS treatment	24 h and 28 days following the injection, days -1-4 and days 26-27 post-treatment for SPT	SPT, FST	↓intake of sucrose solution, context-dependent changes in the FST	(352)
BALB/c mice	male	0.33 mg/kg	Escherichia coli (O127:B8)	IP	acute LPS treatment	24 h, 48 h, 72 h following the injection	FST, TST, LMA	↑immobility time in the FST, ↑immobility time in the TST, ↓locomotor activity	(194)
CD-1 mice	male	0.001 and 0.005 mg/mouse	Escherichia coli (O26:B6)	IP	acute LPS treatment	120-144 min following the injection	TST, FST, OFT	↑immobility time in the TST, ↑floating time in the FST, ↓central, peripheral, and total line crossings in the OFT, ↓number of rears in the OFT	(230)
ICR mice	male	0.83 mg/kg	not specified	IP	chronic (16 days) LPS treatment	11-16 days following the injections	NSFT, SPT, FST	↑immobility time in the FST, ↓climbing time in the FST, ↓sucrose preference, ↑latency to feed	(177)
C57BL/6J mice	male	0.83 mg/kg	Escherichia coli (O127:B8)	IP	acute LPS treatment	2 h, 24 h following the injection	LDB, SPT, OFT	↓exploratory locomotor activity, ↓time spent in the central area of the arena, ↑thigmotaxis, ↓time spent exploring the illuminated side of the LDB, ↓sucrose preference	(196)
C57BL/6J mice	male	0.33 mg/kg	Escherichia coli (O127:B8)	IP	acute LPS treatment	24 h following the injection	LMA, concurrent choice task	↓nose pokes, ↓horizontal activity	(182)
NMRI mice	male	0.31, 0.63 and 1.25 mg/kg	Escherichia coli (O55:B5)	IP	acute LPS treatment	2 h, 6 h, 24 h following the injection	FST, TST, OFT, stress-induced hyperthermia test, SPT	↓locomotor activity, dose-and time-dependent effects in the FST and TST, ↓sucrose preference,	(192)
CD1 and C57Bl/6J mice	male	0.83 mg/kg	Escherichia coli (O127:B8)	IP	acute LPS treatment	24 h, 27 h following the injection	SPT, FST	↑immobility time in the FST, ↓sucrose preference	(189)
CD1 mice	male	0.83 mg/kg	Escherichia coli (O127:B8)	IP	acute LPS treatment	6 h, 24 h, 48 h, 72 h, 96 h following the injection	SPT, TST, FST, LMA	↑immobility time in the FST, ↑immobility time in the TST, ↓sucrose consumption, ↓number of line crossings, ↓number of rearings	(184)
BALB/c mice	male	0.33 mg/kg	Escherichia coli (O127:B8)	IP	acute LPS treatment	2, 4, 8, 12, 24, 39 h following the injection	SPT, SIT	↓social exploration, ↓sucrose preference	(187)
CD1 mice	male	0.83 mg/kg	Escherichia coli (O127:B8)	IP	acute LPS treatment	6, 24, 28 h following the injection	TST, FST, LMA	↑immobility time in the FST, ↑immobility time in the TST, ↓locomotor activity	(185)
BALB/c mice	male	0.0003-0.005 mg/mouse	Escherichia coli (O127:B8)	IP	acute LPS treatment	2, 5, 10, 24, 48 h following the injection	FST, LMA, SIT, EPM	↓locomotor activity, ↑immobility time in the FST, ↓social interaction, ↓novel object exploration, no effects on anxiety in the OFT and EPM	(186)
ICR mice	male	0.1, 0.3 and 0.5 mg/kg	Escherichia coli (O127:B8)	IP	acute LPS treatment	24 h following the injection	LMA, LDB, HBT	↓time spent in the illuminated section in the LDB, ↓number of head dips in the HBT, no effects on locomotor activity	(181)
		0.5, 1 and 3 mg/kg		IT	acute LPS treatment	24 h following the injection			
Swiss mice	male	0.5 mg/kg	Escherichia coli (O55:B5)	IP	chronic (10 days) LPS treatment	24 h following the injection	TST, FST, OFT, HBT, EPM	no effects on locomotor activity, no effects on self-grooming behavior, ↑immobility time in the FST, ↓climbing time in the FST, ↑immobility time in the TST, ↓percentage of entries into the open arms in the EPM, ↓percentage of time in the open arms in the EPM, ↓head dips in the HBT	(195)

IT, intratracheal; SPT, sucrose preference test/saccharin preference test; FST, forced swim test; OFT, open field test; TST, tail suspension test; EPM, elevated plus maze test; LDB, light–dark box test; ICSS, intracranial self-stimulation; LMA, locomotor activity test; MWM, Morris water maze test; CPP, conditioned place preference; NSFT, novelty suppressed feeding test; SIT, social interaction test/social exploration test; HBT, hole−board test.

↑ increase; ↓ decrease; – no change.

**Table 6:** Experimental endotoxemia in rats.

Animals	Sex	LPS dose	Serotype	Injection site	Experimental design	Analysis time after injection	Behavioral tests	Behavioral outcomes	References
Sprague–Dawley rats	male	0.01 and 0.1 mg/kg	Escherichia coli (0111:B4)	IP	acute LPS treatment	2 and 6 h following the injection	FST	no effect on the development of the immobility	(292)
Sprague–Dawley rats	male	0.6 mg/kg	Escherichia coli (O55:B5)	IP	chronic (56 days) and acute LPS treatment	20 h following the last injection	OFT, FST	↓locomotor activity, ↑immobility in the FST	(353)
Sprague–Dawley rats	male	0.5 mg/kg	not specified	IP	every other day with a total of 4 times	not specified	SPT, FST, OFT, EPM, Y maze test	↑immobility in the FST, ↓struggling time in the FST, ↓sucrose preference index, no effect on the total ambulatory distance, frequency to the center, mean speed, rearing, and grooming times in the OFT, no effect on the duration in the close arm in the EPM, and the preference index of the novel arm in the Y maze	(197)
Sprague–Dawley rats	male	0.05, 0.1, 0.2 mg/kg	Escherichia coli (O55:B5)	IP	acute LPS treatment	3 h following the injection	FR5/chow choice task	↓lever pressing in the FR5/chow choice task	(183)
Wistar rats	male	1 mg/kg	not specified	IP	acute LPS treatment	26 h following the injection	SPT, FST, OFT	↓sugar water preference rate, ↓numbers of horizontal and vertical movements in the OFT, ↑immobility time in the FST	(354)
Sprague–Dawley rats	male	1 mg/kg	Escherichia coli (0111:B4)	IP	acute LPS treatment	18-24 h following the injection	FST, OFT	↓total distance traveled in the OFT, ↑immobility time in the FST	(329)
Wistar rats	male	0.05 mg/kg	Escherichia coli (0111:B4)	IP	acute LPS treatment	6-22 h following the injection	SPT, OFT	↓sucrose preference, ↓spontaneous locomotor activity	(355)
Wistar rats	male	0.02 and 0.08 mg	Escherichia coli (O55:B5)	ICV	acute LPS treatment	40 weeks following the last injection	OFT, EPM, FST, MWM	↓distance traveled and the number of rearing events in the OFT, ↓the percentage of time spent in the open arms and the number of entries into the open arms in the EPM, ↑the immobility time in the FST, ↓the swimming time and climbing time in the FST, ↑the latency to reach the visible platform and the total distance traveled in the MWM during training, ↓the number of target crossings and time spent in the target quadrant in the MWM during probe trial	(207)
Long-Evans rats	male	0.5, 1, 2 mg/kg	Escherichia coli (O26:B6)	IP	acute LPS treatment	24 h after the last injection for several days	progressive ratio task	↓breakpoint	(356)
		0.1, 0.5, 1, 2, 5 mg/kg/week		SC	chronic (14 days) LPS treatment			no effect on breakpoint	
		0.0105 mg/week		ICV	chronic (14 days) LPS treatment			↓breakpoints	
Sprague–Dawley rats	male	0.31, 0.63 and 1.25 mg/kg	Escherichia coli (O55:B5)	IP	chronic (5 days) and acute LPS treatment	2 h, 6 h, 24 h, 48 h, 72 h, 96 h following the injection	SPT, FST, OFT	↓locomotor activity in the OFT, ↓sucrose preference, ↑immobility time in the FST	(193)
Wistar rats	male	0.83 mg/kg	Escherichia coli (O55:B5)	IP	acute LPS treatment	24 h following the injection	SPT, FST, OFT, splash test	↑immobility time in the FST, ↓sucrose preference, ↓time of grooming, ↑latency to grooming, no effect in the number of crossings in the OFT	(320)
Sprague–Dawley rats	male	0.1 mg/kg	Escherichia coli (0111:B4)	IP	acute LPS treatment	24 h and 48 h following the injection	SPT, LMA	↓saccharin consumption, ↓saccharin preference, ↓locomotor activity	(323)
Sprague–Dawley rats	male	0.075 or 0.3 mg//kg/day	Escherichia coli (O55:B5)	osmotic minipumps implanted in the abdomen	chronic (1 or 4 weeks) LPS treatment	day 6 (for the 1-week experiment), and day 24 (for the 4-weeks experiment)	FST, OFT	no effects in the OFT and FST	(200)
Wistar rats	male	0.05, 0.1 and 0.2 mg/rat	Escherichia coli (0111:B4)	IP	acute LPS treatment	1.5 h following the injection	ICSS	↓ICSS during the ascending sequence of current presentations, no effects on responding to a series of descending currents	(357)
Sprague–Dawley rats	male	0.5 mg/kg	not specified	IP	chronic (every other day, 7 times) LPS treatment	not specified	SPT, FST, OFT	↓sucrose preference, ↑immobility time in the FST, ↓number of crossings and rearings in the OFT	(199)
Sprague–Dawley rats	male	0.001, 0.005, 0.015, 0.05, 0.125 and 0.25 mg/kg	Escherichia coli (0111:B4)	IP	acute LPS treatment	2 h, 4 h, 24 h following the injection	SIT, LMA, SPT	↓social interaction, ↓locomotor activity, ↓saccharin preference	(209)
Wistar rats	not specified	0.05 mg/kg	Escherichia coli (O127:B8)	IP	acute LPS treatment	90 min following the injection	CPP, sucrose consumption test, LMA	↓sucrose intake, no effects on the sucrose-induced place preference, no effects on locomotor activity	(358)
Sprague–Dawley rats	male	0.1 mg/rat	Escherichia coli (O55:B5)	IP	chronic (four injections per week every other day) and acute LPS treatment	90 min following the injection	ICSS	↓ICSS responding upon acute exposure to LPS	(221)
Wistar rats	male	0.1 mg/kg	Escherichia coli (O26:B6)	IP	acute LPS treatment	2, 4, 6, 13 and 24 h following the injection	SPT	↓sucrose consumption	(191)
Fisher 344 rats	male	0.01, 0.05, 0.2, 1 mg/kg	Escherichia coli (O55:B5)	IP	acute LPS treatment	2, 4, 6, 13, 24, 48, 72 h following the injection (depending on the test)	SPT, OFT, SIT, sexual behavior assessment	↓saccharin preference, ↓saccharin consumption, ↓sexual behavior, ↓social exploration, ↓number of line crossings and rearings in the OFT	(190)

SPT, sucrose preference test/saccharin preference test; FST, forced swim test; OFT, open field test; TST, tail suspension test; EPM, elevated plus maze test; LDB, light–dark box test; ICSS, intracranial self-stimulation; LMA, locomotor activity test; MWM, Morris water maze test; CPP, conditioned place preference; NSFT, novelty suppressed feeding test; SIT, social interaction test/social exploration test.

↑ increase; ↓ decrease; – no change.

### 4.1 Experimental endotoxemia in rodents

After intraperitoneal administration of LPS to rats, endotoxin appears in the systemic circulation within 15 minutes, reaching peak levels after 90 minutes. The amount of endotoxin in the systemic circulation represents approximately 10% of the injected dose (178). The administration of LPS to rodents triggers the activation of both the immune and neuroendocrine systems. Following LPS injection, ACTH peaks in the blood after 30 minutes via the intravenous route and after 90 minutes via the intraperitoneal route. Among proinflammatory cytokines, TNF-α exhibits the most rapid increase, reaching peak plasma concentrations within 1 hour (intravenous) and 1.5 hours (intraperitoneal). Following intravenous administration, IL-6 and IL-1β peak concurrently at 1.5 hours. In contrast, after intraperitoneal injection, IL-1β levels show minimal change, while IL-6 peaks later, at approximately 2.5 hours (179).

The elevation of proinflammatory cytokines following LPS administration exerts a significant influence on rodent behavior. Beyond inducing sickness behavior, LPS administration elicits depressive-like behavior, characterized by increased immobility in the TST and reduced sucrose preference (180), as well as anxiety-like behavior (181). Furthermore, LPS administration impairs motivation, diminishing the willingness to exert effort for reward (182, 183).

The acute inflammatory phase, characterized by a pronounced peak in proinflammatory cytokines, is thought to subside approximately 6 hours post-LPS administration in rodents. Coinciding with this decline, sickness behavior begins to wane, and depressive-like behaviors become more prominent. By 24 hours post-LPS administration, locomotor activity normalizes, and mice exhibit depressive-like behavior including increased immobility in the TST and FST, as well as decreased sucrose consumption (184–186). This timeframe is also associated with the manifestation of anhedonia-like behavior, as corroborated by a reduction in sucrose preference (187–189).

However, these observations are not universally consistent. Many studies report a temporal overlap between sickness and depressive-like behaviors, complicating their clear dissociation. Furthermore, sickness behavior can persist beyond the 24-hour mark. For instance, in rats, LPS administration results in concurrent reductions in locomotor activity and saccharin preference at 4 hours post-injection. While saccharin preference normalizes by 13 hours, deficits in food consumption and body weight can persist for up to 72 hours (190). In this instance, depressive-like and sickness behaviors not only develop concurrently, but indicators of sickness persist beyond the resolution of depressive-like behavior. A parallel pattern is reported in a separate study, wherein reduced sucrose consumption recovers within 24 hours of LPS administration, while deficits in body weight and food intake remain evident beyond this time point (191). This raises a critical methodological question: can a reduction in sweet solution consumption or preference, when observed concurrently with sickness behavior, be reliably interpreted as a specific indicator of anhedonia-like behavior? For instance, the decrease in sucrose preference observed in mice 24 to 72 hours post-LPS is accompanied by a parallel reduction in total fluid intake (a sign of sickness behavior), confounding the interpretation of the hedonic deficit (192). Biesmans et al. demonstrate that, by 24 hours post-LPS administration, proinflammatory cytokine levels and locomotor activity return to baseline, whereas total fluid intake remains suppressed. The authors argue that anhedonia-like behavior emerges only on days 2–3 post-administration. By this later time point, sickness behavior has fully resolved and total fluid intake has normalized, yet a deficit in sucrose preference persists (193). Sickness behavior exhibits greater persistence in aged animals. For instance, aged mice display sustained reductions in locomotor activity and feeding at both 24 and 48 hours post-LPS administration. Concurrently, these animals show increased immobility in the FST, indicating a temporal overlap between prolonged sickness behavior and the manifestation of depressive-like behavior (194). The administration of LPS in rodents reliably induces immune activation and sickness behavior, however, the consequent depressive-like state, particularly anhedonia, is frequently temporally confounded with sickness, presenting a significant methodological challenge for its specific assessment.

#### 4.1.1 LPS doses and administration regimens

**Figure 1:**
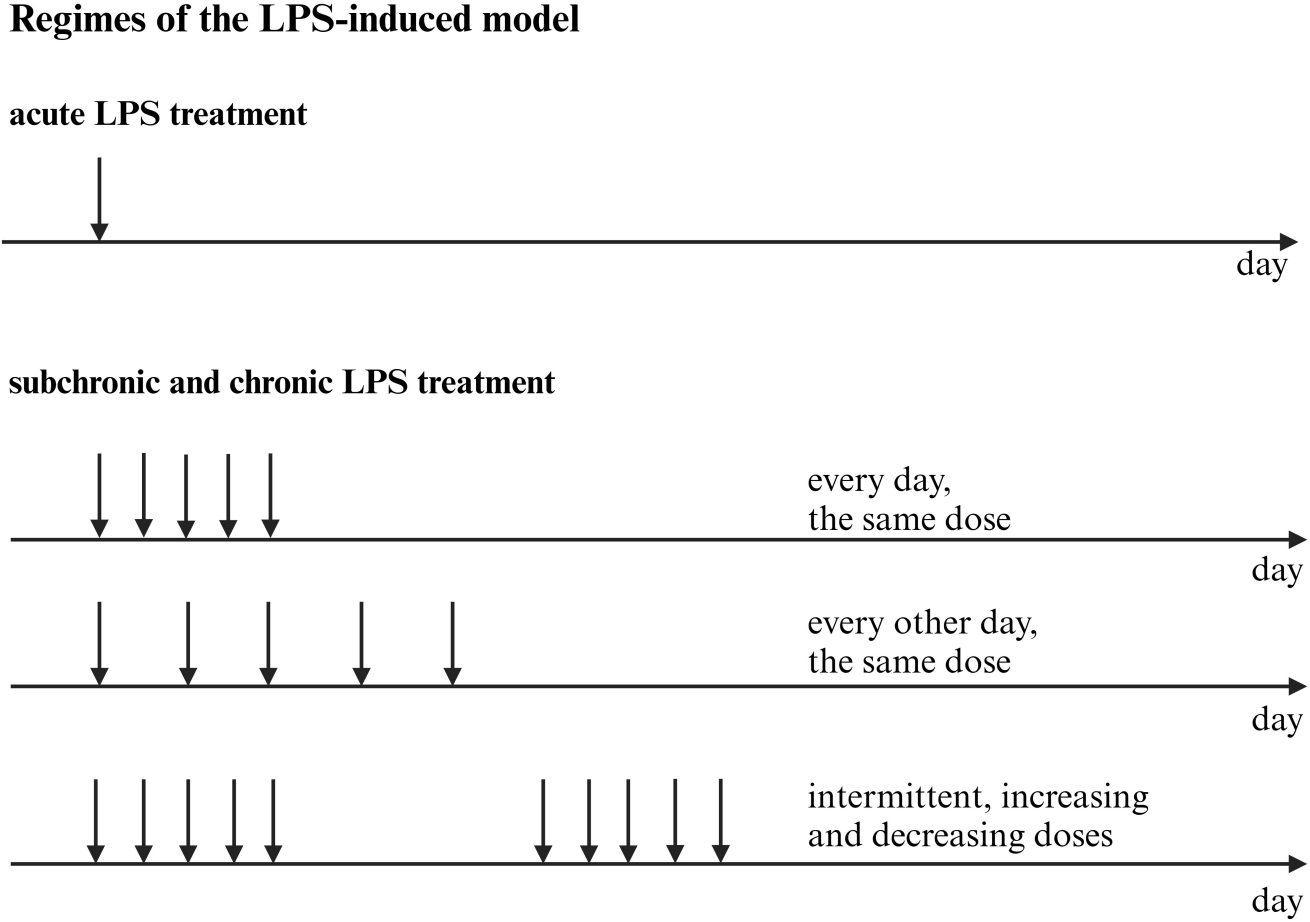
LPS administration regimens. There are three main regimens of LPS administration: acute, subchronic and chronic. In acute administration, LPS is administered once in a certain dose. In subchronic and chronic administration, LPS is administered repeatedly in the same dose or in different doses. The most commonly used administration options are: every day at the same dose, every other day at the same dose, and intermittent administration of increasing and decreasing doses.

LPS administration regimens in rodents can be categorized into two principal types. The majority of studies employ an acute LPS administration. The second category encompasses various subchronic and chronic protocols, which may involve repeated administration of a consistent dose over several days, intermittent dosing (e.g., every other day), or schedules with incrementally escalating and de-escalating doses (see **Figure 1**).

Mice subjected to ten consecutive daily intraperitoneal LPS injections at a fixed dose display depressive- and anxiety-like behavior (195). Similarly, a 16-day chronic LPS regimen leads to attenuated body weight gain and the emergence of a depressive-like behavior, characterized by increased immobility in the FST and reduced sucrose preference (196). An analogous behavioral outcomes observed in rats receiving four LPS injections administered every other day (197). Furthermore, mice exposed to eight intermittent LPS injections (every other day) develop a depressive-like state, as corroborated by increased immobility in both the FST and TST (198). In rats, LPS administered every other day over a two-week period induces a depressive-like behavior, evidenced by reduced sucrose preference and increased immobility in the FST. This behavioral shift is accompanied by elevated mRNA expression of the pro-inflammatory cytokines IL-1β, IL-6, and TNF-α in the frontal cortex and hippocampus (199). Notably, these studies report that animals do not develop tolerance to repeated LPS administration. The mechanisms underlying this lack of tolerance are not understood, and the failure of immune and behavioral responses to diminish during chronic LPS exposure suggests this phenomenon is insufficiently characterized. Chronic LPS administration via osmotic minipumps fails to induce a depressive-like behavior, and sickness behavior (e.g., reduced food intake and body weight) is observed only transiently during the first five days before all parameters normalize (200).

Interval LPS administration using variable doses represents an attempt to establish a chronic model while circumventing the development of tolerance. For instance, female mice receiving escalating and de-escalating LPS doses over five-day periods, repeated at one-month intervals for four consecutive months, develop a chronic depressive-like state. This is evidenced by increased immobility in the FST and decreased sucrose preference (201). Under the three-month LPS administration regimen, sucrose preference decreased only during the initial three escalating doses, normalizing during the subsequent de-escalating doses and remaining at baseline until the next injection series. A stable anhedonia-like behavior emerged only after the final (fourth) series of injections and persisted for the ensuing seven weeks. Notably, this same administration regime fails to induce anhedonia-like behavior in male mice (202).

While less commonly employed than intraperitoneal injection, central administration of LPS via the intracerebroventricular route is an effective method for directly engaging brain immune signaling. This approach reliably induces sickness behavior, anxiety-like, and depressive-like states in rodents. These phenotypes are validated through a constellation of behavioral outcomes: decreased body weight and sucrose preference (anhedonia), increased immobility in the FST and reduced time spent in the center of the open field (203–205). These behavioral alterations are paralleled by a pronounced neuroinflammatory response within the hippocampus, characterized by upregulated mRNA expression of IDO, TNF-α, IL-6, and iNOS (206). Depressive-like behavior is observed even 40 weeks after icv LPS administration, indicating the long-term nature of the behavioral changes (207). In ICE (Interleukin-1 beta converting enzyme, caspase-1) knockout mice, depressive-like behavior is induced by systemic (intraperitoneal) administration of LPS but not by central (intracerebroventricular) administration. This dissociation suggests that distinct neuroimmune mechanisms underlie the behavioral response to peripherally versus centrally delivered LPS (204). Collectively, the evidence demonstrates that direct activation of brain immune pathways is sufficient to precipitate a depressive-like state. Future research dissecting the distinct molecular signatures and behavioral outcomes elicited by central versus peripheral immune challenges (e.g., with LPS or cytokines) is crucial to elucidate the precise contribution of neuroinflammation to the pathogenesis of depression-like states.

According to the review, the C57BL/6 mouse strain is the most frequently employed in the LPS-induced model of depressive-like behavior. The predominant protocol involves a single intraperitoneal LPS injection, most commonly at a dose of 0.83 mg/kg. The LPS used is typically derived from Escherichia coli serotypes, with O55:B5, O127:B8, and 0111:B4 being the most prevalent. Behavioral assessments most commonly include the FST, TST, OFT, and SPT (208).

In studies utilizing rats, the Sprague-Dawley strain is most frequently employed. The acute intraperitoneal injection of LPS constitutes the most common administration route. The effective LPS dose range in rats is typically lower (0.05–0.1 mg/kg) than that used in murine models. The lipopolysaccharide is most often derived from Escherichia coli serotypes O55:B5 and 0111:B4. Behavioral outcomes are primarily assessed using the OFT, SPT, and FST.

The majority of studies employ relatively high LPS doses. These doses prolong sickness behavior and induce a significant reduction in locomotor activity. This suppression of general activity can, in turn, confound performance in the FST, a common assay for depressive-like behavior. Importantly, even low doses of endotoxin are capable of inducing measurable physiological alterations. Bison et al. evaluated the effects of intraperitoneal LPS administration across a dose range of 0.001, 0.005, 0.015, 0.05, 0.125, and 0.25 mg/kg. The study demonstrated a dose-dependent hierarchy of immune and neuroendocrine activation: a dose of 0.015 mg/kg elevated ACTH; 0.005 mg/kg increased corticosterone, IL-1β, and IL-6; and even the lowest dose (0.001 mg/kg) elevated TNF-α. Significant behavioral alterations were observed at all tested doses, beginning with the lowest. Social interaction, body weight, and saccharin preference were suppressed across the entire dose range, while locomotor activity was reduced at doses of 0.005 mg/kg and higher. These results clearly indicate that immune and neuroendocrine system activation, alongside measurable behavioral changes, occur at very low LPS doses (209). This finding raises a critical question regarding the justification for employing high LPS doses to model depression. This is also supported by the possibility of significant differences in gene expression profiles in immune cells at different LPS doses. Transcriptome analysis showed that in mouse monocytes, low-dose LPS (100 pg/mL) preferentially induces inflammatory interferon-responsive genes, while high-dose LPS (1 µg/mL) suppresses certain subsets of inflammatory genes and upregulates exhaustion signatures with metabolic and proliferative pathways (210).

The LPS-induced model of depressive-like behavior in rodents is characterized by considerable methodological heterogeneity, including variations in species, strain, LPS dose, behavioral tests, and assessment timelines. This variability complicates cross-study comparisons and obscures the identification of fundamental mechanisms underlying the depressive-like phenotype. Disparities in experimental conditions can substantially influence outcomes, sometimes yielding directly contradictory results. Importantly, heterogeneity arises not only from methodological differences but also from intrinsic biological variation within subject populations. While immune system activation occurs in nearly all animals following an inflammatory challenge, a depressive-like state develops only in a subset. This stratification into susceptible and resilient subgroups is well-established in models like CUMS. However, such pre- or post-LPS administration stratification is rarely applied in inflammatory models, despite evidence that individual animals exhibit differential vulnerability to inflammation-induced depressive-like behavior (211, 212).

A notable issue is the frequent lack of concordance between behavioral parameters measured by different tests, even within a single study. For instance, effects may be evident in the FST but absent in the SPT (213). Conversely, a decrease in sucrose preference may be observed without corresponding changes in the FST (192, 214). This inconsistency underscores that these behavioral assays likely capture distinct or only partially overlapping animal states, rather than a unified “depressive-like” construct.

The LPS-induced model is a valuable but imperfect tool. Methodological inconsistencies, confounding effects from high doses, a lack of concordance between behavioral assays, and a failure to account for individual biological variation limit the clarity of the mechanisms it reveals. Future research requires more standardized, low-dose approaches and a focus on stratifying subjects by vulnerability to better elucidate the molecular mechanisms linking inflammation to depressive-like behavior.

#### 4.1.2 Sensitivity to endotoxin in rodents

**Figure 2:**
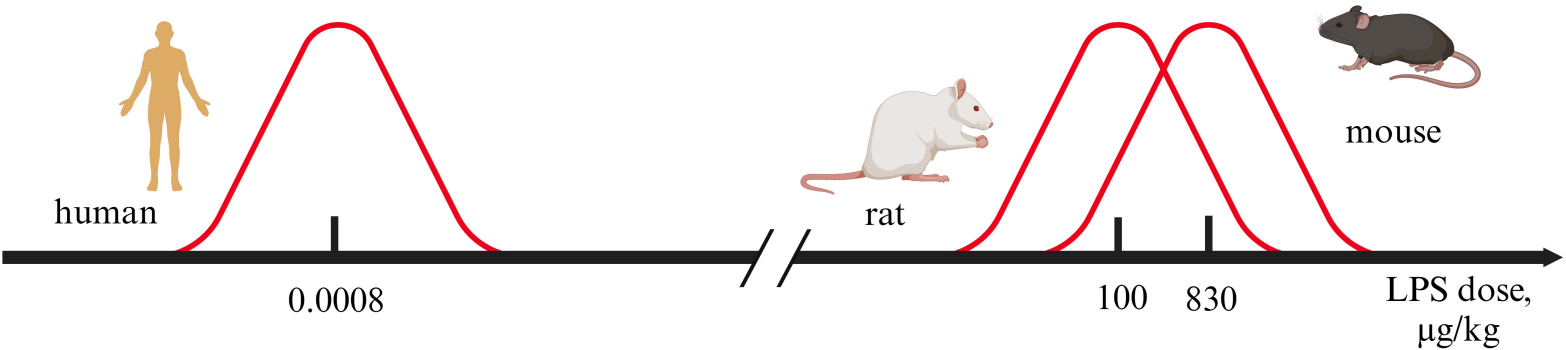
Comparative LPS doses across species. The LPS doses required to elicit a systemic inflammatory response differ by 100,000 to 1,000,000 times between species, with typical doses being 0.8 ng/kg in humans, 100 µg/kg in rats, and 830 µg/kg in mice.

The doses of LPS employed in experimental endotoxemia differ drastically between humans and rodents. In rodent models, administered doses typically range from 0.01 to 8 mg/kg, with the most frequent doses being 0.1 mg/kg in rats and 0.83 mg/kg in mice (see **Figure 2**). In contrast, human studies use doses in the ng/kg range, representing a 100,000 to 1,000,000-fold lower dose than those administered to rodents. The requirement for higher LPS doses in rodent models is attributed to their inherent lower sensitivity to endotoxin. A leading hypothesis posits that this differential sensitivity is mediated by species-specific serum proteins. Supporting this, *in vitro* evidence demonstrates that mouse serum suppresses LPS-induced TNF-α production in human monocytes more potently than human serum. This suppressive effect is both concentration-dependent, increasing with higher serum content in the medium, and sensitive to trypsin digestion (215). Comparative transcriptomic analyses reveal profound evolutionary divergence in the innate immune response to LPS. A key distinction emerges between LPS-sensitive species (e.g., rabbit, pig, sheep, cow, chimpanzee, human) and LPS-resistant species (e.g., mice, rats, baboon, rhesus). Notably, sensitive species exhibit a primed immune state, characterized by elevated basal expression of LPS-responsive genes even in the absence of stimulation. In contrast, following LPS challenge, resistant species are characterized by the upregulation of genes that mediate LPS detoxification, diminish bacterial growth, and involved in autophagy and apoptosis. These data indicate fundamentally different organizational strategies in the innate immune systems across species (216).

A direct comparison reveals a difference in potency: to elevate plasma IL-6 to 1000 pg/ml, humans require an endotoxin dose of 2 ng/kg, whereas mice require 500 ng/kg (217). Thus, to achieve an equivalent physiological response, mice necessitate a 250-fold higher dose. It is crucial to emphasize that the required differential is 250-fold, not the 100,000 to 1,000,000-fold higher doses commonly administered in rodent research. This discrepancy directly challenges the validity of employing high doses in experimental models. While the conserved nature of the vertebrate inflammatory response supports a degree of translational relevance, this profound difference in LPS sensitivity underscores fundamental organizational disparities between the human and rodent immune systems.

#### 4.1.3 Endotoxin tolerance

A principal challenge in developing a chronic inflammatory model of depression is the induction of tolerance upon repeated LPS administration, whereby inflammatory and neuroendocrine responses diminish with each subsequent dose. This phenomenon is well-documented. In rats, intraperitoneal LPS administration over several days leads to a desensitization of the HPAA, characterized by a blunted ACTH and corticosterone response to a subsequent LPS challenge (218, 219). Similarly, in mice, administering the same LPS dose over three consecutive days results in a markedly attenuated increase in plasma levels of TNF-α, IL-1β, and IL-6 compared to an acute single administration, confirming the development of immune tolerance. Notably, this preconditioning effect can have long-term consequences, conferring protection against the detrimental effects observed in a subsequent experimental sepsis model even nine months later (220).

Repeated LPS administration influences not only immune and neuroendocrine parameters but also behavior, including depressive-like behavior. Compared to chronic regimens, acute LPS administration induces a more pronounced reduction in body weight and sucrose preference, alongside a greater increase in immobility in the FST (16). In rats, acute LPS elevates the threshold currents required for intracranial self-stimulation, indicating an anhedonia-like state. In contrast, subchronic LPS administration leads to the development of tolerance by day 7, which is accompanied by the restoration of normal ICSS parameters (221).

The development of tolerance is an evolutionarily conserved process, documented in both rodents and humans. In humans, administering endotoxin at a dose of 2 ng/kg daily for 5 days induces tolerance, evidenced by a progressive decline in cytokine levels. By the fifth day, concentrations of IL-10, TNF-α, IL-6, and IL-1RA are reduced by approximately half compared to day one. This blunted cytokine response is accompanied by a corresponding attenuation of sickness symptoms (165).

While the development of tolerance has been well-documented in the peripheral immune system, its occurrence within the CNS remains unclear. Evidence suggests a dissociation between peripheral and central inflammatory responses. For instance, mice preconditioned with a low LPS dose fail to show the expected decreases in immobility in the FST or in the expression of TNF-α, IL-6, and IL-1β in the frontal cortex, hippocampus, and striatum following a subsequent high-dose LPS challenge. This indicates a lack of central tolerance to LPS following this preconditioning regimen (222). Similarly, animals subjected to a subchronic regimen of gradually increasing LPS doses twice daily for 9.5 days do not exhibit elevated serum levels of TNF-α, IL-6, and IL-1β compared to animals receiving a single acute injection. However, mRNA levels of TNF-α and IL-1β in the brain remain significantly elevated. These data further indicate the development of peripheral tolerance without concurrent CNS tolerance (223).

Results from a separate study indicate the development of CNS tolerance, though its dynamics differ from those in the periphery. In mice, a single LPS injection induces a pronounced increase in blood levels of IL-1β, TNF-α, IL-6, IL-12, and IFN-γ, but only a marginal elevation of these cytokines in the brain. A second LPS injection elicits a markedly attenuated peripheral cytokine response compared to the first, confirming peripheral tolerance. In contrast, this same challenge provokes a significant increase in brain cytokine levels, accompanied by microglial activation. Notably, a decrease in central cytokine concentrations occurs only after the fourth LPS injection, demonstrating the eventual development of CNS tolerance. These findings suggest a capacity for immune memory within microglial cells (224). Mice pre-exposed to a low LPS dose over four days exhibit reduced levels of TNF-α and IL-1β in the substantia nigra. Furthermore, when these preconditioned animals are subsequently challenged with a high LPS dose, they fail to show the expected increases in TNF-α and IL-1β in this region and do not display microglial activation. These results demonstrate that low-dose LPS preconditioning effectively attenuates subsequent high-dose LPS-induced neuroinflammation, as evidenced by suppressed proinflammatory cytokine production and inhibited microglial reactivity (225).

The state of tolerance, while reversible, can persist for several weeks. The underlying mechanisms remain poorly understood but are hypothesized to involve epigenetic and transcriptional reprogramming. Specifically, it is proposed that tolerance arises from modifications in gene expression and the activity of key signaling pathways. Putative mediators include histone modifications, chromatin remodeling, DNA methylation, and the actions of microRNAs and long non-coding RNAs (226, 227). Elucidating the mechanisms of tolerance is of significant scientific interest. A detailed understanding of how this process unfolds in both the periphery and CNS could provide the means to disrupt it, thereby enabling the development of robust, chronic inflammatory models of depression.

### 4.2 Cytokine-induced models of depression

The cytokine-induced model of depressive-like behavior is used less frequently than the LPS model. It is based on the direct administration of proinflammatory cytokines (IL-1β, TNF-α, IL-6) to experimental animals (see **Table 7**). Protocols typically involve an acute cytokine injection, and behavioral effects are assessed within a short post-injection window, during the acute phase of the inflammatory response.

**Table 7:** Cytokine-induced models of depression in rodents.

Animals	Sex	Cytokine	Dose	Injection site	Experimental design	Analysis time after injection	Behavioral tests	Behavioral outcomes	References
Sprague Dawley rats	male	IL-1β	1, 2 and 4 μg/kg	IP	acute injection	90 min after injection	FR5/chow-feeding procedure	↓lever pressing	(228)
Wistar rats	not specified	IL-1β	4 μg/kg	IP	acute injection	90 min after injection	CPP, sucrose consumption test, LMA	↓sucrose intake, ↓locomotor activity, no effects on the sucrose-induced place preference	(358)
CD-1 mice	male	IL-1β	0.1, 0.3 and 1 μg/mouse	IP	acute injection	90-102 min after injection	TST, FST, OFT	↑immobility time in the TST, ↑floating time in the FST, ↓locomotor activity, ↓number of rears	(230)
C57BL/6 x 129/Sv mice	male	IL-1β	5 µg/kg/day	SC	chronic injection (4 weeks)	1 and 4 weeks after injection	SPT, SIT	↓sucrose preference, ↓social exploration	(233)
Long-Evans rats	male and female	IL-1β	2 µg/kg	IP	acute injection	3 h, 24 h, 48 h, 72 h after injection	progressive ratio schedule of reinforcement	↓break point for responding for sucrose	(229)
Sprague Dawley rats	male	IL-1β	0.1 µg/rat	ICV	acute injection	2 h after injection	sucrose consumption test, SIT, TST	↓sucrose intake, ↓social interaction, ↑immobility time in the TST	(231)
Sprague Dawley rats	male	IL-1β	0.01 µg/rat/day	ICV	chronic (14 days) injection	after 14 days	OFT, SPT, EPM	↓number of rears, ↑defecation, ↓time spent in the open arm, ↑time spent in the closed arm, ↓sucrose preference	(232)
CD-1 mice	male	IL-1β	0.025, 0.05, 0.1, 0.4, 0.8 μg/mouse	IP	acute injection	45 min and 24 h after injection	chocolate milk consumption	↓consumption of the chocolate milk	(243)
		IL-6	0.1, 0.2, 0.8, 1.6 μg/mouse			45 min and 24 h after injection		no effects on consumption of the chocolate milk	
		TNF-α	1, 2, 4 μg/mouse			30 min and 24 h after injection		↓consumption of the chocolate milk	
Wistar rats	male	IL-2	0.25, 0.5 and 1 µg/rat	IP	acute injection	15 min, 24 h, 1 and 2 weeks after injection	ICSS	↓number of reinforced responses, ↑reward threshold	(242)
		IL-1β	1 and 2 µg/rat					no effects	
		IL-6	1 µg/rat					no effects	
Sprague Dawley rats	male	IL-6	2, 4, 6 and 8 μg/kg	IP	acute injection	45 min after injection	FR5/chow-feeding procedure	↓lever pressing	(241)
Swiss mice	male	TNF-α	1х10^-13^ - 1х10^-7^ µg/site	ICV	acute injection	60 min after injection	TST, FST, OFT, SPT	no effects on the number of crossings in the OFT, ↑immobility time in the TST, ↑immobility time in the FST, ↓sucrose intake	(239)
Swiss mice	female	TNF-α	1х10^-12^ µg/mouse	ICV	acute injection	60 min after injection	TST, OFT	no effects on locomotor activity, ↑immobility time in the TST	(235)
Swiss mice	male	TNF-α	1х10^-12^ and 1х10^-10^ µg/site	ICV	acute injection	60 min after injection	TST, FST, LMA	↑immobility time in the TST, ↑immobility time in the FST, no effects on locomotor activity	(236)
Swiss mice	female	TNF-α	1х10^-12^ µg/site	ICV	acute injection	30 min after injection	TST, OFT	no effects on locomotor activity, ↑immobility time in the TST	(234)
Swiss mice	female	TNF-α	1х10^-12^ µg/site	ICV	acute injection	30 min after injection	TST, OFT	no effects on locomotor activity, ↑immobility time in the TST	(237)
Swiss mice	female	TNF-α	1х10^-12^ µg/site	ICV	acute injection	30 min after injection	TST, OFT	no effects on locomotor activity, ↑immobility time in the TST	(238)
Sprague Dawley rats	male	TNF-α	1 μg/24 h	ICV	chronic (14 days) injection	after 14 days	FST	↑immobility time in the FST	(240)

SPT, sucrose preference test/saccharin preference test; FST, forced swim test; OFT, open field test; TST, tail suspension test; EPM, elevated plus maze test; ICSS, intracranial self-stimulation; LMA, locomotor activity test; CPP, conditioned place preference; SIT, social interaction test/social exploration test.

↑ increase; ↓ decrease; – no change.

For example, intraperitoneal IL-1β administration in rats reduces the willingness to work for food, indicating motivational deficits (228). This decrease in motivation is observed 3 hours post-injection in males and persists for up to 48 hours in females (229). In mice, IL-1β injection increases immobility in the FST and TST after 1.5 hours, concurrent with reductions in locomotor activity, food intake, and body weight, illustrating the temporal overlap of depressive-like and sickness behaviors (230). Similarly, acute icv IL-1β administration in rats suppresses social behavior and sucrose consumption within 2 hours (231). Collectively, these studies demonstrate that cytokine-induced depressive-like behavior develops during the acute phase of the inflammatory response.

Chronic administration protocols also elicit depressive-like behavior. In rats, chronic icv infusion of IL-1β increases anxiety-like behavior in the open field and elevated plus maze tests and reduces sucrose preference (232). Similarly, chronic subcutaneous IL-1β administration in mice decreases sucrose preference; notably, this effect emerges only after the first week of treatment, as preference in the experimental group does not initially differ from controls, underscoring the necessity of a chronic administration scheme (233).

The central administration of TNF-α reliably induces depressive-like behavior in rodent models. In mice, a single icv TNF-α injection increases immobility in the TST within 30–60 minutes, an effect not attributable to general motor impairment, as locomotor activity in the open field remains unchanged (234–238). Furthermore, icv TNF-α administration increases immobility in the FST and reduces sucrose preference (239). This effect is also observed with chronic TNF-α administration, evidenced by increased immobility in the FST (240).

Administration of IL-6 to rats induces a specific motivational dysfunction. This is evidenced by a decrease in lever pressing for a preferred food (high-carbohydrate pellets) and a concurrent increase in consumption of a less-preferred substitute (standard lab chow). Importantly, IL-6 does not alter food intake or preference in parallel free-feeding choice studies, indicating that the effect is not due to a generalized suppression of appetite or a shift in preference for the high carbohydrate pellets (241).

The specific behavioral effects induced by proinflammatory cytokines are likely determined by their unique spectra of biological activity. For example, a single intraperitoneal injection of IL-2 in rats induces an anhedonia-like state, as measured by an elevated threshold for intracranial self-stimulation. This effect is observed at 15 minutes, 24 hours, and even one week post-administration - a pattern not elicited by IL-1β or IL-6 (242). Furthermore, in mice, acute administration of IL-1β and TNF-α, but not IL-6, transiently reduces the consumption of highly palatable chocolate milk within 30–45 minutes, with consumption normalizing by 24 hours (243).

As the presented data indicate, proinflammatory cytokines are frequently administered via the icv route. This method directly models central neuroinflammation without the confound of peripheral immune activation. The resulting depressive-like behaviors are typically observed during the acute phase of the response, are short-lived, and resolve rapidly.

### 4.3 IFN-α-induced model of depression

The administration of IFN-α for treating chronic hepatitis C in humans can induce depressive symptoms. To model this condition preclinically, rodents are administered IFN-α (see **Table 8**). Depressive-like behavior can be elicited by both acute (iv or icv) and chronic (sc) IFN-α regimens. Chronic subcutaneous administration is notably more potent; achieving an equivalent increase in TST immobility requires a threefold lower dose compared to intravenous administration (244). Both acute and chronic iv IFN-α administration in rodents induces depressive-like behavior in the FST without affecting general locomotor activity (245, 246). However, findings from acute administration protocols are less consistent. For instance, in rats, sucrose consumption is reduced when recombinant IFN-α is administered 20 minutes before testing, but not at 40 or 80 minutes (247). Acute IFN-α does not induce anhedonia-like behavior in female rats, as measured by intracranial self-stimulation thresholds (248). Furthermore, acute IFN-α in mice decreases, rather than increases, immobility in the FST and has no effect in the TST (249).

**Table 8:** IFN-α-induced model of depression in rodents.

Animals	Sex	IFN-α dose	Injection site	Experimental design	Analysis time after injection	Behavioral tests	Behavioral outcomes	References
C57BL/6J mice	male	0.06–6 MIU/kg	SC	chronic (7 days) IFN-α treatment	not specified	SPT, FST, OFT, TST	↑immobility time in the TST, ↑immobility time in the FST, no effects on locomotor activity, ↓sucrose preference	(250)
Swiss albino mice	male	0.4–1.6 IU/kg	SC	chronic (5-15 days) IFN-α treatment	24 h after the last IFN-α injection	FST, OFT	↑immobility time in the FST, no effects on locomotor activity	(253)
BALB/c mice	male	60,000 U/kg	IP	chronic (8 days) IFN-α treatment	15 h after the last IFN-α injection	FST, TST, EPM	↑immobility time in the FST, no effects on immobility time in the TST, no effects on the percentage of time spent in the open arms and the number of open arm entries	(359)
ddY mice	male	2 x 10^6^ – 2 x 10^7^ I.U./kg	IV	acute IFN-α treatment	15 min after IFN-α injection	TST, LMA	↑immobility time in the TST, no effects on locomotor activity	(244)
		6 x 10^4^ I.U./mouse	ICV	acute IFN-α treatment	15 min after IFN-α injection		↑immobility time in the TST	
		6 x 10^4^ – 6 x 10^6^ I.U./kg	SC	chronic (7 days) IFN-α treatment	30 min after the last IFN-α injection		↑immobility time in the TST	
C57Bl/6 mice	male	8 IU/kg	SC	chronic (8 days) IFN-α treatment	24 h after the last IFN-α injection	SPT, FST, LMA, hot plate test, formalin test	no effects on locomotor activity, ↑immobility time in the FST, ↓sucrose preference, no effects on latency to respond in the hot plate test, ↑nociceptive behavior in the formalin test	(252)
ddY mice	male	600–60 000 IU/kg	IV	acute and chronic (7 days) IFN-α treatment	15 h after the last IFN-α injection	FST, LMA	no effects on locomotor activity, ↑immobility time in the FST	(245)
BALB/c mice	male	60,000 U/ml/kg	IP	chronic (14 days) IFN-α treatment	before and after two weeks of IFN-α injections	object exploration test, FST, TST	no effects on locomotor activity, ↑immobility time in the FST, ↑immobility time in the TST, ↓explorative behavior	(212)
CD1 mice	male	6, 60 or 600 µg/kg	IP	two weekly IFN-α treatments	different	SPT, FST	no effects on sucrose preference and immobility time in the FST	(257)
C57BL/6J mice	male	2 × 10^6^ IU/kg	IP	acute IFN-α treatment	2 h after IFN-α injection	FST, TST, EPM, LDB	no effects in TST, EPM and LDB, ↓immobility time in the FST	(249)
C57BL/6J mice	male	4 × 10^5^ IU/kg	IP	chronic (4-5 weeks) IFN-α treatment	after 5 weeks of IFN-α injections	FST, TST	↑immobility time in the TST, ↑immobility time in the FST	(251)
C57BL/6J mice	male	1 × 10^5^ or 4 × 10^5^ IU/kg	IP	chronic (2, 4, 5 weeks) IFN-α treatment	after 2 and 5 weeks of IFN-α injections	FST, TST, hot plate test, rotarod test, OFT, LDB, EPM, crawley’s sociability and preference for social novelty test	↑immobility time in the TST, ↑immobility time in the FST, no effects in hot plate test, rotarod test, OFT, LDB and EPM, ↓social interactions	(254)
CD-1 mice	male	100 and 1000 IU	ICV	acute and chronic (6 days) IFN-α treatment	over a six-day period	LMA, sucrose consumption test	no effects on locomotor activity, ↓sucrose consumption	(360)
Lister hooded rats	male	10^4^ units/kg	IP	chronic (33 days) IFN-α treatment	every third day	three-bottle (1%, 8%, and 32%) sucrose consumption test	↑intake of the 32% sucrose solution, ↓intake of the 1% sucrose solution	(293)
Lewis rats	male and female	650 µg/wk PEG-2a or PEG-2b	IP	chronic (3 weeks) IFN-α treatment	every week	FST, LMA	no effects on locomotor activity and immobility time in the FST	(256)
Wistar rats	male	6 x 10^2^ – 6 x 10^4^ IU/kg	IV	acute and chronic (7 days) IFN-α treatment	15 min after IFN-α injection	FST, LMA	↑immobility time in the FST, no effects on locomotor activity	(246)
Sprague-Dawley rats	male	6 x 10^4^ IU/kg	SC	chronic (7 days) IFN-α treatment	24 h after IFN-α injection	SPT, FST, OFT	no effects on locomotor activity, ↑immobility time in the FST, no effects on saccharin preference	(213)
Long–Evans rats	female	10 and 1000 units/kg	IP	acute IFN-α treatment	15 min after IFN-α injection and twice weekly over 4 weeks	brain stimulation reward, LMA	no effects in frequency thresholds, no effects on locomotor activity	(248)
Wistar rats	male	100,000 IU/kg	IP	acute and chronic (14 days) IFN-α treatment	2 h after IFN-α injection (acute version) and 15 min after the last IFN-α injection (chronic version)	sucrose pellet self-administration, FST	no effects on sucrose pellet self-administration, no effects in FST	(255)
Wistar rats	male	170000 IU/kg	SC	chronic (4 weeks, three times per week) IFN-α treatment	after 4 weeks of IFN-α injections	FST, SPT, EPM	↓time in the open arms, ↑time in the closed arms, ↓entries into open arms, ↑floating time in the FST, ↓saccharin preference after four weeks of IFN-α administration, ↑saccharin preference in repeated saccharin preference testing	(295)

SPT, sucrose preference test/saccharin preference test; FST, forced swim test; TST, tail suspension test; EPM, elevated plus maze test; LMA, locomotor activity test; OFT, open field test; LDB, light–dark box test.

↑ increase; ↓ decrease; – no change.

Chronic IFN-α administration in mice reliably induces a depressive-like behavior, characterized by increased immobility in the FST and TST and decreased sucrose preference (250–252). The time course for the onset of this phenotype varies across studies. While some report the emergence of depressive-like behavior within 5 to 15 days of treatment (253), others observe significant effects in the FST and TST only after 5 weeks, with no changes detected at earlier time points (254).

However, the induction of depressive-like behavior by peripheral IFN-α administration is not universally replicated. For instance, chronic IFN-α administration in rats fails to elicit sickness or depressive-like behaviors, as indicated by unchanged IL-6 and TNF-α levels, stable body weight and locomotor activity, and unaltered immobility in the FST (255, 256). Similarly, in mice, IFN-α administration has been reported to have no effect on immobility in the FST or sucrose preference (257).

The heterogeneity of experimental animal populations is a potential contributor to the poor reproducibility of the IFN-α-induced model of depressive-like behavior. Supporting this, chronic IFN-α administration in mice elevates TNF-α levels uniformly across all subjects, but a depressive-like phenotype, as measured by increased immobility in the FST, develops only in a susceptible subgroup (212).

Most studies employ chronic IFN-α administration regimens or directly compare different regimens and routes. Consequently, this model offers a distinct advantage: the capacity to replicate the persistent behavioral alterations characteristic of clinical depression.

### 4.4 BCG-induced model of depression

An alternative method for inducing depressive-like behavior in animals utilizes the administration of the tuberculosis vaccine, bacille Calmette-Guérin (258). The Bacille Calmette-Guérin (BCG) vaccine is a live, attenuated strain of Mycobacterium bovis, a bacterium related to Mycobacterium tuberculosis which elicits a complex and prolonged immune response involving both innate and adaptive immune systems. Following BCG administration, mice exhibit a sustained proinflammatory state. TNF-α and IFN-γ remain elevated for up to three weeks, while IL-6 shows an initial increase during the first week followed by a decline. The acute behavioral response includes transient sickness: locomotor activity decreases 8–24 hours post-injection and recovers by 48 hours. Body weight loss is also observed, with recovery occurring only 11 days post-injection. Subsequently, from day 7 to day 21, a depressive-like behavior emerges, characterized by increased immobility in the FST. Single-point measurement reveals reduced sucrose consumption on day 21, daily monitoring shows a decrease only during the first two days, with consumption normalizing thereafter (259). Findings across studies reveal variability in the time course of BCG-induced effects. In one study, BCG inoculation reduces sucrose preference on day 11 and impairs social interaction by day 13 (260). Some researchers report that food intake and body weight normalize within 3–4 days, and locomotor activity recovers by day 5, with depressive-like behavior (increased immobility in the FST and TST) emerging on day 7 (261). Conversely, other researchers observe that depressive-like behavior develops only by day 14, after the complete resolution of sickness symptoms such as reduced body weight and locomotor activity (262). The temporal dynamics of sickness behavior following BCG administration are likely influenced by dosage and age. Higher vaccine doses prolong body weight loss and reduced locomotor activity (263). Age is also a critical factor: adult animals recover locomotor activity by day 7, whereas aged animals require up to 14 days. Similarly, BCG-induced deficits in sucrose preference and increased immobility in the TST resolve in adult mice by day 14 but persist in aged mice beyond 21 days post-injection (264). Furthermore, individual susceptibility varies significantly within a cohort. While sickness behavior develops uniformly, an increase in TST immobility manifests only in a susceptible subgroup. Approximately 30% of mice are resilient to these depressant effects (211).

The BCG model is established as a method for inducing a chronic depressive-like state in rodents. A key proposed advantage of this model is its capacity to temporally dissociate acute sickness behavior from subsequent, persistent depressive-like behavior. The standard protocol involves a single intraperitoneal BCG injection (10^6^–10^8^ CFU/mouse) in male mice (see **Table 9**). In the majority of studies, depressive-like behavior emerges in the period from 7 to 21 days post-administration, after the resolution of sickness behavior. The temporal manifestation of sickness and depressive-like behaviors in this model closely parallels the clinical trajectory observed in patients undergoing IFN-α therapy (62). In both cases, an initial phase dominated by sickness symptoms is followed by a subsequent phase characterized by depressive symptomatology. However, a critical question remains unresolved: do sickness behavior and depressive-like behavior constitute distinct, sequential phases, or are they concurrent and overlapping states?

**Table 9:** BCG-induced model of depression in rodents.

Animals	Sex	BCG dose	Injection site	Experimental design	Analysis time after injection	Behavioral tests	Behavioral outcomes	References
CD-1 mice	male	1.44×10^7^–1.536×10^8^ CFU/mouse	IP	acute BCG treatment	6-7 days after BCG treatment	LMA, TST	↓locomotor activity in BCG-susceptible mice, ↑immobility time in the TST in BCG-susceptible mice	(211)
Balb/c mice	male	10^8^ CFU/mouse	IP	acute BCG treatment	1, 7, 14 and 21 days after BCG treatment	LMA, TST, SPT	↓locomotor activity, ↓number of rearings, ↓sucrose preference, ↑immobility time in the TST	(264)
BALB/c mice	male	10^9^ CFU/mouse	IP	acute BCG treatment	24 h, 7, 14 and 21 days after BCG treatment	FST, LMA, TST, wheel running activity test	↓spontaneous locomotor activity, ↓voluntary wheel running activity, ↑immobility time in the TST, ↑immobility time in the FST	(262)
CD1(ICR) mice, B6.129-Indo^tm1Alm^/J (IDO^-/-)^ or C57BL/6J mice	male	10^8^ CFU/mouse	IP	acute BCG treatment	baseline and 1-7 days after BCG treatment	FST, LMA, TST	↓locomotor activity, ↑immobility time in the TST, ↑immobility time in the FST	(261)
CD1 mice	male	10^7^ CFU/mouse	IP	acute BCG treatment	depending on the behavioral parameter from baseline to 21 days after BCG treatment	FST, LMA, TST, SPT, wheel running activity test	↓locomotor activity, ↑immobility time in the TST, ↑immobility time in the FST, ↓sucrose intake, ↓voluntary wheel running	(259)
C57BL/6J mice, CD1(ICR) mice, B6.129S7-Ifngr1^tm1Agt^/J	male	10^6^-10^8^ CFU/mouse	IP	acute BCG treatment	depending on the behavioral parameter from baseline to 7 days after BCG treatment	FST, LMA, TST	↓locomotor activity, ↑immobility time in the TST, ↑immobility time in the FST	(263)
C57BL6/J mice	male	3×10^7^ CFU/mouse	IP	acute BCG treatment	11-13 days after BCG treatment	SPT, SIT	↓sucrose preference, ↓social interaction	(260)

SPT, sucrose preference test/saccharin preference test; FST, forced swim test; TST, tail suspension test; LMA, locomotor activity test; SIT, social interaction test/social exploration test.

↑ increase; ↓ decrease; – no change.

This model further elucidates the pathophysiological mechanisms underlying depression. A comparative transcriptomic analysis of microglia and peripheral macrophages from control and BCG-challenged mice elucidates potential mechanisms contributing to the development of depressive-like behavior and delineates the specific role of neuroinflammation. Among more than 15,000 genes tested, 562 and 3,851 were differentially expressed between the BCG-challenged and control groups in microglia and peripheral macrophages, respectively. The substantially lower number of transcriptional changes in microglia suggests a more tightly regulated, rapidly resolving inflammatory response within the central nervous system or significantly distinct response mechanisms to the inflammatory stimulus (265).

The BCG model is established as a method for inducing a chronic depressive-like state with the valuable feature of temporally dissociating acute sickness from subsequent depressive-like behavior. It thus provides a critical platform for investigating the mechanisms of prolonged, inflammation-driven depression.

### 4.5 Poly (I:C)-induced model of depression

Poly(I:C) is a synthetic double-stranded RNA that mimics viral infection by activating toll-like receptor 3 and inducing the inflammatory response. In rodent models, systemic administration of poly(I:C) via the intraperitoneal route reliably induce sickness behavior, as evidenced by decreased food intake, weight loss, and suppressed locomotor activity (266–268). Animals exhibit increased plasma levels of IL-6, TNF-α, and IL-10, as well as increased corticosterone levels (269). The classic sickness phenotype—reduced food intake and body weight loss—is observed following poly(I:C) administration via both peripheral (intraperitoneal) and central (intracerebroventricular) routes (270). Beyond the acute sickness phenotype, poly(I:C) also induces depressive-like behavior and is therefore used to model depressive-like states.

A primary application of poly(I:C) is in the experimental model of prenatal maternal immune activation. There is a lot of evidence that maternal infection during gestation can cause irreversible changes in the fetal brain that may subsequently lead to behavioral alterations and increase the risk of occurrence of neuropsychiatric disorders in adulthood. Administration of poly(I:C) to pregnant females reliably induces depressive-like behavior in the offspring. However, findings regarding the sex-specificity of these effects remain inconsistent. While some studies report the emergence of such behaviors exclusively in female offspring (271), others document significant alterations only in males (272, 273).

Beyond its application in prenatal maternal immune activation models, poly(I:C) is also administered systemically to adult male rodents. A single intraperitoneal injection in rats induces 1) sickness behavior, characterized by reduced locomotor activity and body weight loss; 2) depressive-like state, evidenced by anhedonia-like behavior (e.g., reduced saccharin preference) or increased immobility time in the FST; and 3) anxiety-like behavior in the open field test (274, 275).

LPS and poly(I:C) may elicit distinct behavioral responses, a critical consideration for experimental design. These immunostimulants have been shown to have different effects not only on the sickness and anxiety-like behaviors of pregnant dams but also on the development of their offspring. A key distinction is that poly(I:C), but not LPS, administration induces delay in growth and sensorimotor development in pups (276). Comparative studies indicate that poly(I:C) is less potent than LPS in inducing sickness behavior. For instance, the dose of poly(I:C) required to elicit fever, anorexia, and lethargy of comparable magnitude to that induced by LPS was greater than the LPS dose by a factor of approximately 50 (267). Notably, a second poly I:C challenge either 1 or 3 weeks later after the initial challenge, fails to induce behavioral tolerance (266). The comparatively lower potency of poly(I:C) and its failure to induce behavioral tolerance upon repeated administration represent potential advantages over LPS for modeling states of prolonged low-grade inflammation. However, a systematic, direct comparison of these immunostimulants—encompassing dose-response relationships, temporal cytokine profiles, and long-term neuroimmune and behavioral outcomes—is required to definitively establish their respective utilities and limitations in chronic inflammatory models.

### 4.6 Inflammatory markers in chronic stress rodent models of depression

An important question is whether depressive-like behavior in non-inflammatory rodent models is associated with concomitant alterations in peripheral inflammatory markers. In the widely used CUMS model, chronic stress induces a significant elevation in circulating CRP levels in rats (277, 278) and increases blood concentrations of pro-inflammatory cytokines, including TNF-α, IL-1β, IL-6, and IFN-γ (177, 277, 279, 280). Collectively, these findings indicate that long-term stress in the CUMS paradigm promotes a state of systemic inflammation (281).

Similarly, the chronic social defeat stress model results in elevated blood levels of TNF-α, IL-6, and IFN-γ (282–284), though data on CRP in this model are lacking. While the mechanisms driving depressive-like behavior in these stress models are undoubtedly complex, the pathogenic role of inflammation is supported by interventional studies. Systemic administration of neutralizing antibodies to deplete specific cytokines (TNF-α or IL-6) attenuates the severity of depressive-like symptoms in both models (285, 286). This preclinical evidence aligns with clinical data linking specific circulating inflammatory mediators to particular depressive symptom profiles. Consequently, these observations suggest that modeling approaches designed to experimentally replicate chronic inflammatory profiles may enhance the validity and specificity of rodent models of depression.

## 5 Face validity

Rodent and human models of inflammation-associated depression differ significantly. These differences extend beyond the dose and route of LPS administration to encompass the assessment timeline and methods used to quantify the depressive-like state. In human experimental endotoxemia, the state is assessed via subjective self-report questionnaires and clinical rating scales. In contrast, preclinical rodent models rely on objective, instrumentally measured behavioral endpoints.

The FST and TST tests are standard assays for assessing depressive-like behavior, yet their validity is questionable for several reasons. Classically, increased immobility in the FST is interpreted as behavioral despair (287). However, the direct relevance of this construct to core clinical symptoms of depression - such as low mood and anhedonia - remains unclear. Alternative interpretations suggest that immobility reflects a coping or adaptation strategy rather than a state of despair (288–291). A more fundamental issue is that the FST/TST themselves constitute severe, acute physical and psychological stressors. These acute stress responses can profoundly confound behavioral readouts. Imagine that you have the flu and a fire breaks out in your house. In all likelihood, despite your illness, you will attempt to escape the burning building. Conversely, if you fall ill aboard a cruise ship that is sinking, you are not likely to accept the situation passively. Most probably, you would strive to swim to the nearest land. The presence of an immediate, severe threat can profoundly alter behavioral priorities, overriding pre-existing states such as sickness or malaise.

In certain instances, the anticipated effects on behavior in the FST are not observed. For example, research indicates that LPS administration in rats does not significantly alter either the time spent actively swimming or the duration of immobility during the test (292).

Behavioral outcomes measured by different tests often lack concordance; for instance, depressive-like behavior may be evident in the FST but absent in the SPT. The reasons for this discrepancy are unclear but likely indicate that these tests assess distinct aspects of the animals’ behavior. A representative example is chronic IFN-α administration in rats, which increases immobility in the FST but does not alter saccharin preference (213). This raises a critical methodological question: which test more validly assesses a depressive-like state? In the specific case of consumption/preference tests, results can be influenced by procedural variables such as the concentration (sweetness) of the solution or the frequency of testing. Notably, chronic IFN-α administration in rats decreases consumption of a 1% sucrose solution while increasing consumption of a 32% solution (293). The observation that chronic IFN-α administration suppresses consumption of a 1% sucrose solution while facilitating intake of a 32% solution strongly parallels the effects of dopamine receptor antagonists reported by Muscat et al. (294). This parallel suggests that the IFN-α effect is more consistent with an alteration in reward sensitivity or palatability than with a generalized drive for increased caloric intake. Nonetheless, a strictly hedonic interpretation does not fully exclude a potential contributory role for caloric factors. The outcome of the saccharin preference test in the IFN-α model is critically dependent on the testing schedule. When administered as a single endpoint measurement after four weeks of IFN-α treatment, rats exhibit a decreased preference for saccharin. Conversely, when the test is conducted repeatedly during the chronic administration period, animals demonstrate an increase in saccharin preference (295).

**Figure 3:**
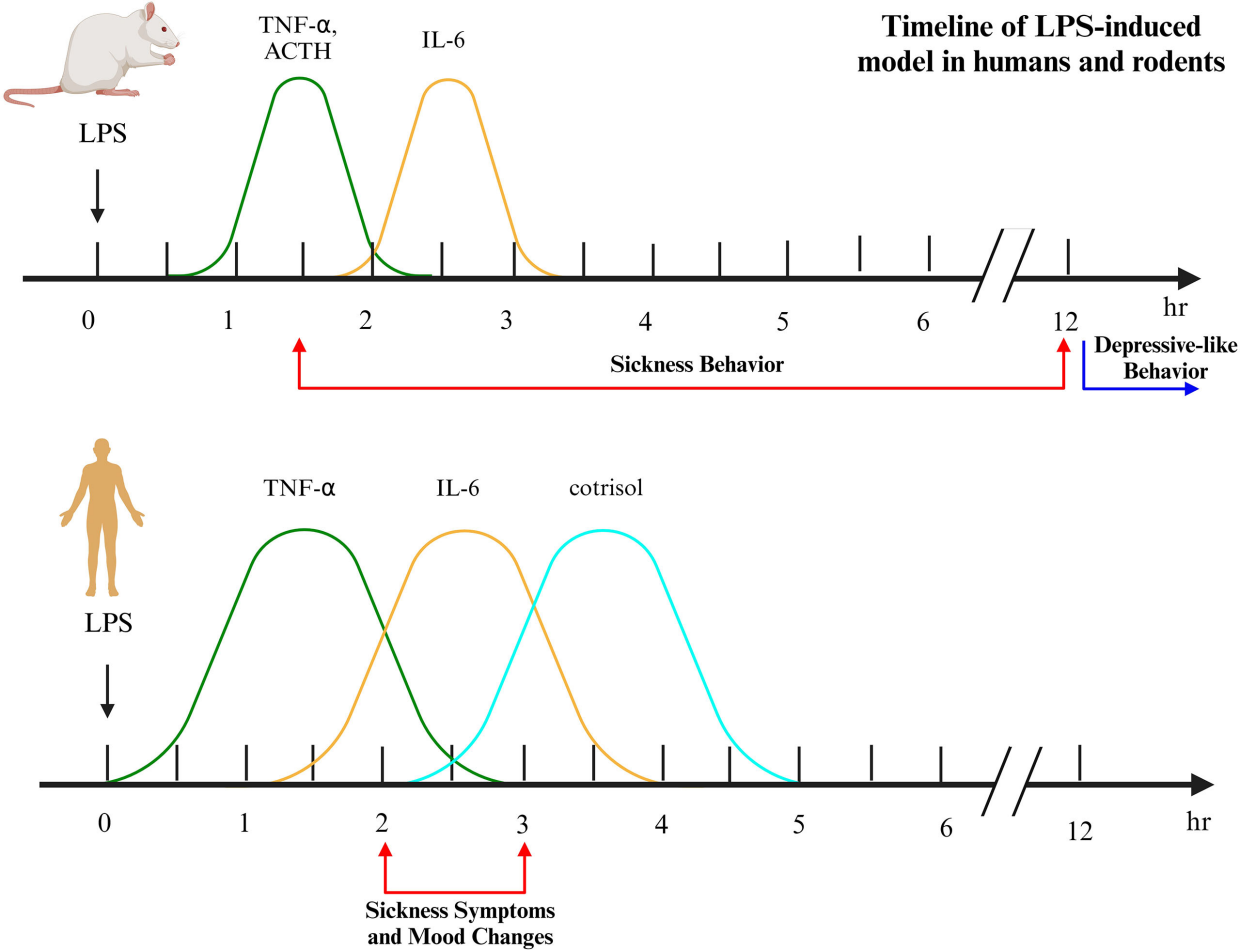
Comparative time course of the behavioral and physiological effects of LPS administration in humans and rodent models. While the temporal elevation of cytokine levels following LPS administration is comparable between humans and rodents, the manifestation of sickness and depressive-like behaviors differs markedly in its chronology. In humans, behavioral changes are transient, with symptoms of sickness and depressed mood emerging concurrently. In contrast, rodent models typically exhibit a more prolonged sickness phase, with depressive-like behaviors appearing after a delayed interval, a temporal dissociation that remains a subject of debate. These interspecies discrepancies may be attributable to the vastly different LPS doses employed, as well as inherent physiological variations in immune and endocrine system organization.

The temporal relationship between inflammation and depressive symptoms is interpreted differently across species. In rodents, depressive-like behavior is typically assessed 24 hours post-LPS, a time point chosen to avoid confounding acute sickness behavior (184, 185). In humans, mood deterioration occurs within 3 hours of LPS administration, coinciding with sickness symptoms, and generally resolves by 6 hours, with few studies evaluating effects beyond 12 hours. Given that both sickness and depressive-like behaviors are initiated by a similar inflammatory cascade, and assuming comparable dynamics of the peripheral immune response in rodents and humans, a critical question arises: why is the presumed onset of inflammation-induced depressive-like behavior temporally dissociated between species (see **Figure 3**)? This temporal dissociation suggests that the molecular mechanisms that trigger development of depressive-like behavior may differ between humans and rodents.

In the LPS-induced model of depression, somatic or neurovegetative symptoms such as reduced food intake, body weight loss, and decreased locomotor activity are robustly and consistently replicated. In contrast, behaviors interpreted as anhedonia are not assessed in all studies and their reproducibility is variable. The subjective, affective symptoms of depression such as low mood, guilt, or worthlessness are inherently unmeasurable in animal models.

Furthermore, the validity of using locomotor activity as a definitive marker for the resolution of sickness behavior is questionable. Locomotor activity is not a direct measure of immune system activation but a behavioral consequence of elevated cytokine levels. This interpretation is complicated by the fact that altered psychomotor activity, either agitation or retardation, is itself a diagnostic symptom of major depressive disorder in humans (296). This distinction necessitates careful translational interpretation: while reduced locomotor activity in rodents is a nonspecific indicator of sickness behavior, in humans, the same phenotype constitutes a diagnostic criterion for depression.

This same interpretive ambiguity applies to feeding behavior and its derived parameters, such as reduced food intake and body weight loss. In humans, significant changes in appetite and weight are diagnostic criteria for depression. In animal models, however, the recovery of these measures is routinely used to confirm the resolution of sickness behavior.

Consequently, the most objective criterion may be the normalization of circulating proinflammatory cytokine levels, which provides direct evidence that the acute systemic inflammatory phase has ended. This biochemical marker could serve as a temporal benchmark to distinguish sickness behavior from depressive-like behavior, if such a clear distinction exists. In humans mood disturbances and neurovegetative symptoms emerge concurrently following an inflammatory challenge. This temporal co-occurrence suggests that attempts to rigidly separate sickness and depressive-like behaviors in rodents may constitute an interpretative error.

Behavioral testing inherently stresses animals, introducing variability from individual differences in stress reactivity. This manifests as a well-established phenotypic dichotomy, segregating rodents into stress-susceptible and stress-resilient subpopulations, even under tightly controlled conditions. This segregation into susceptible and resilient phenotypes is particularly evident in the assessment of anhedonia-like behavior (297–299). Consequently, the absence of pre-experimental stratification can obscure treatment effects, as outcomes are diluted in mixed cohorts. This methodological confound likely contributes to poor reproducibility. To enhance data consistency, we propose incorporating baseline behavioral assessments to stratify subjects, thereby reducing within-group variance and increasing experimental sensitivity. Animals may be stratified into distinct behavioral subgroups based on baseline measures, such as locomotor activity. Subjects categorized in this manner subsequently demonstrate significant differences in the forced swim test (300–302) and sucrose preference test (303).

A further critical consideration for modeling depression and interpreting experimental data is the high comorbidity between depression and anxiety disorders. Anxiety disorders are concurrently diagnosed in approximately 71.7% of patients with MDD (304). The precise role of systemic inflammation in the pathogenesis of anxiety disorders remains poorly understood. While elevated CRP levels have been reported in men with anxiety, this association is not observed in women. Furthermore, neither IL-6 nor TNF-α levels correlate consistently with anxiety (305), and meta-analyses have revealed no significant differences in inflammatory cytokine levels between healthy controls and patients with anxiety disorders (306, 307). It is important to note that the existing body of research measuring proinflammatory cytokines in anxiety remains limited. Consequently, while the evidence for a direct inflammatory signature in generalized anxiety is currently weak, a contributory role for inflammation in specific anxiety phenotypes cannot be definitively excluded. As with depression, it is plausible that elevated proinflammatory cytokine levels may characterize only a subset of patients with anxiety (308).

Depression can be clinically subtyped into anxious and non-anxious forms. The anxious subtype is typically characterized by greater overall severity (309) and a poorer response to first-line antidepressant pharmacotherapy (310, 311). In rodent models, inflammatory challenges consistently induce a mixed behavioral phenotype encompassing both depressive-like and anxiety-like behaviors. A methodological inconsistency arises because anxiety is not uniformly assessed across studies utilizing these models. When anxiety-like behavior is not measured, it remains ambiguous whether the model simulates an anxious or non-anxious depressive subtype.

The validity of common behavioral tests is undermined by stress confounds, lack of concordance, and the conflation of sickness with depressive symptoms. The fundamental difference in symptom timing between species suggests either divergent molecular mechanisms or errors in interpretation. To advance the field, researchers must integrate more objective biomarkers (e.g., cytokines) and, crucially, account for individual biological variation through pre-experimental stratification and baseline behavioral assessment. Furthermore, incorporating standardized assessments of anxiety-like behavior into the experimental design of inflammatory depression models is essential. This practice would allow for the characterization of the specific depressive subtype being modeled, thereby greatly improving the interpretability of behavioral data and the translational relevance of findings, particularly for evaluating the efficacy of novel or existing antidepressant drugs.

## 6 Predictive validity

### 6.1 Antidepressant effects in clinical trials and inflammatory models of depression

Most contemporary antidepressant drugs are designed to restore monoaminergic neurotransmission, primarily by inhibiting neurotransmitter reuptake or monoamine oxidase activity. Beyond these canonical mechanisms, a growing body of evidence indicates that antidepressants possess significant anti-inflammatory properties. Various classes of antidepressants, including SSRIs, monoamine oxidase inhibitors, and tricyclic antidepressants, can suppress LPS-induced rodent microglial activation *in vitro*. This immunomodulatory effect is characterized by the inhibition of proinflammatory cytokine production (e.g., IL-6, IL-1β, TNF-α) and the reduction of neurotoxic mediators such as NO and reactive oxygen species (312).

The anti-inflammatory properties of antidepressants are well-substantiated by both clinical trials and preclinical studies. This effect is demonstrated by the ability of antidepressant treatment to reduce circulating levels of proinflammatory cytokines in patients with depression (14, 313). Meta-analysis indicate that antidepressant therapy significantly decreases peripheral concentrations of IL-4, IL-6, and IL-10, though it does not alter levels of IL-1β, IL-2, TNF-α, IFN-γ, or CRP (314). Treatment with SSRIs significantly lowers IL-6 and TNF-α in patients with major depressive disorder without affecting CRP levels (315). Similarly, a four-week regimen of duloxetine (a serotonin-norepinephrine reuptake inhibitor) effectively reduces serum concentrations of IL-8, IL-12, and IFN-γ, but not IL-1β, IL-2, IL-6, or TNF-α (316). Collectively, these findings indicate that antidepressants can normalize the levels of a specific subset of cytokines that are often elevated in depression (17, 18).

A critical criterion for assessing the predictive validity of the inflammation-induced depression model is whether clinically effective antidepressants can ameliorate depressive-like behavior within it.

Currently, evidence for antidepressant efficacy in human experimental endotoxemia is limited and not fully convincing. For instance, a one-week pretreatment with bupropion fails to reduce LPS-induced depressive symptoms (134). Conversely, a five-day pretreatment with citalopram attenuates these depressive symptoms without altering peripheral cytokine levels (317).

Preclinical evidence suggests that antidepressants can modulate both the inflammatory response and depressive-like behaviors in the LPS model. Acute pretreatment of rats with fluoxetine reduces blood levels of TNF-α, IL-6, and IL-1β 90 minutes after LPS challenge (318). An anti-inflammatory effect is accompanied by a behavioral one, as acute administration of SSRIs in mice suppresses the LPS-induced rise in TNF-α and reduces immobility in the TST (319). The efficacy of antidepressant pretreatment appears to depend on administration duration. Chronic fluoxetine pretreatment in mice attenuates LPS-induced depressive-like behavior, evidenced by restored sucrose preference and reduced TST immobility (180). Chronic, but not acute, pretreatment with imipramine in rats normalizes LPS-impaired saccharin preference, indicating a reversal of anhedonia-like behavior (190).

The effects of antidepressants are complex and often dissociated. While these drugs reliably reduce proinflammatory cytokine levels, they do not consistently attenuate depressive-like behavior across all behavioral tests. For example, chronic administration of various antidepressants prevents the LPS-induced increase in IL-6, IL-1β, IFN-γ, and TNF-α protein levels in multiple rat brain regions. These same treatments reduce immobility in the FST but fail to improve sucrose preference (320). Similarly, chronic imipramine and venlafaxine administration do not restore sweet milk consumption in IL-1β- or LPS-induced models (321). In aged female mice, an 11-day fluoxetine regimen decreases the LPS-induced expression of hippocampal IL-1β and TNF-α and splenic IL-6, IL-1β, IL-10, and TNF-α. However, this treatment fails to normalize sucrose preference or immobility in the FST (322). In some cases, antidepressants may even induce anhedonic-like effects. Chronic desipramine lowers LPS-induced TNF-α but itself reduces saccharin consumption and preference, an effect also observed, albeit to a lesser degree, with paroxetine (323).

This lack of reproducible behavioral efficacy may be attributable to the predominant use of acute LPS challenge models. In contrast, studies employing chronic paradigms report more consistent effects. In a chronic model using female mice, fluoxetine administered intraperitoneally for one month following the last LPS injection successfully restored sucrose preference (202). These findings were corroborated by another group, which demonstrated that chronic fluoxetine reverses LPS-induced increases in FST immobility and decreases in sucrose consumption (201).

The data reviewed above indicate that clinically used antidepressants can mitigate the severity of LPS-induced depressive-like behavior. However, these effects are not uniformly reproducible across studies, and antidepressant efficacy often varies depending on the specific behavioral test employed. Clinical trial data indicate that antidepressant treatment selectively reduces circulating levels of specific proinflammatory cytokines in patients with depression. This presents a dual interpretation. First, the inconsistent correlation between cytokine reduction and symptom improvement challenges a simple, direct causal link between peripheral inflammation and depressive symptoms. Conversely, the reproducible downregulation of specific cytokines by effective antidepressants suggests that those particular immune mediators may play a pathogenic role in depression.

The glutamate N-methyl-D-aspartate (NMDA) receptor antagonist ketamine exerts rapid antidepressant effects at low doses in both major depression and treatment-resistant depression, offering a distinct alternative to conventional antidepressants (324). Ketamine’s mechanism of action is distinct from that of classic antidepressants and remains incompletely understood. Beyond its primary NMDA receptor antagonism, preclinical and clinical evidence indicates that ketamine also exerts anti-inflammatory effects. This immunomodulation may occur independently of, or in concert with, NMDA receptor blockade, potentially via direct suppression of pro-inflammatory cytokines or indirect modulation of the kynurenine pathway (325). Ketamine administration attenuates depressive-like behavior in the LPS-induced rodent model, as demonstrated by increased sucrose preference and decreased immobility in the FST (326–329). Ketamine administration lowers TNF-α, IL-1β, and IL-6 levels in the hippocampus (328) and prefrontal cortex (327) but not in the periphery (329). Notably, clinical data suggest a potential limitation: among patients with treatment-resistant depression, low-dose ketamine infusion appears effective primarily in those without baseline low-grade inflammation. This indicates that inflammation-associated depressive symptoms may exhibit resistance to ketamine’s antidepressant effects in humans (330).

Classic serotonergic psychedelics, including N,N-dimethyltryptamine (DMT), 5-methoxy-N,N-DMT, and psilocybin, have demonstrated rapid antidepressant potential in clinical trials and are under active investigation (331, 332). These compounds possess notable anti-inflammatory properties, positioning them as promising agents for targeting neuroinflammation (333, 334). Preclinical evidence supports this potential. For instance, psilocybin exhibits antidepressant-like effects in a chronic stress model of depression (335). In a rodent model of systemic inflammation, DMT was shown to attenuate both the cytokine response and depressive-like behavior in the FST induced by a two-week LPS administration (336). However, data on the efficacy of serotonergic psychedelics in inflammation-related models of depression remain limited. Generating such evidence would be invaluable for further evaluating the predictive validity of these preclinical paradigms.

While antidepressants and rapid-acting agents possess clear anti-inflammatory properties and demonstrate efficacy in inflammation-induced rodent models of depression and human clinical trials, their therapeutic effects on depressive-like states and the inflammatory response are not uniform. This inconsistency underscores the complexity of the relationship between depression and inflammation, suggesting that inflammatory pathways are contributory but not solely determinative factors in the pathophysiology of depressive-like states.

## 7 Conclusion

**Figure 4:**
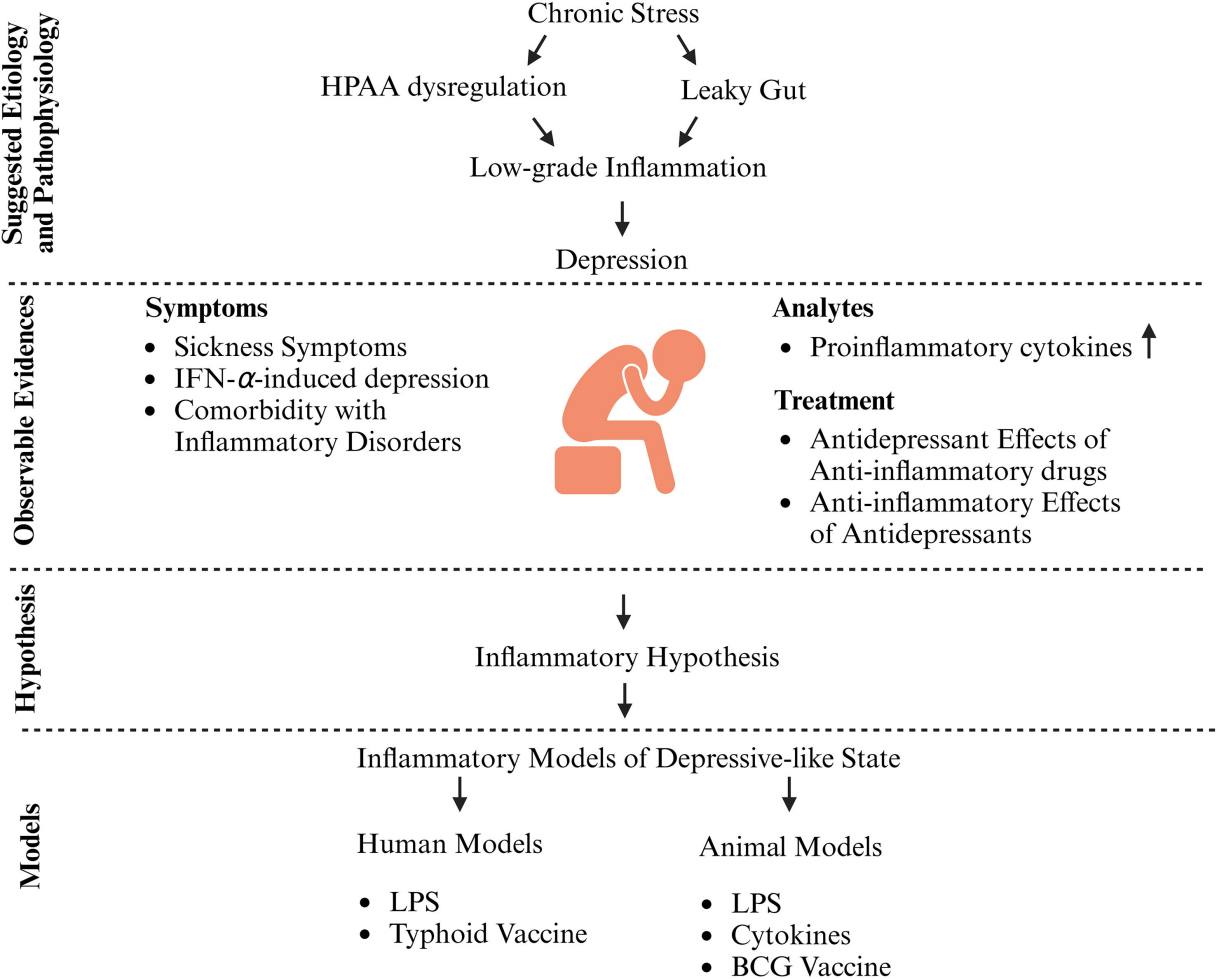
Inflammation and depression. Chronic stress, with its associated elevation in corticosteroids, can dysregulate the HPAA and compromise intestinal barrier function, potentially resulting in a state of low-grade systemic inflammation characterized by elevated pro-inflammatory cytokines. This sustained inflammatory state is hypothesized to contribute to the development of depression. This important role of inflammation is supported by multiple clinical observations: elevated cytokines in depressed patients, significant comorbidity with inflammatory diseases, the symptomatic overlap between sickness behavior and depression, the induction of depressive-like states following IFN-α or LPS administration, and the reciprocal anti-inflammatory properties of some antidepressants and the antidepressant effects of some anti-inflammatory agents. Based on this body of evidence, both human and rodent inflammatory models of depression have been developed. These models are predicated on the administration of immune system activators to reliably induce a depressive-like state.

Clinical evidence supports the role of inflammation in a subset of depressive patients. This includes observations that patients with depression often exhibit elevated blood levels of proinflammatory cytokines, that inflammatory disorders are frequently comorbid with depression, and that exogenously administered cytokines (e.g., IFN-α) can induce depressive symptoms. Furthermore, anti-inflammatory treatments can attenuate depressive symptoms in certain cases, while conventional antidepressants have been shown to exert anti-inflammatory effects. Collectively, these findings indicate that inflammation is involved in the pathogenesis of depression at least in some patients (see **Figure 4**). Consequently, some researchers advocate for the inclusion of an inflammation specifier for major depressive disorder in the Sixth Edition of the Diagnostic and Statistical Manual of Mental Disorders (DSM-6) (337).

The induction of depressive-like state in animals and humans through bacterial endotoxin or proinflammatory cytokine administration aligns with clinical evidence and supports the construct validity of inflammatory models of depression. However, several critical questions remain unresolved. These include the relative contributions of peripheral versus central inflammation to symptom development and their interdependence. In addition, it remains unclear how the brain changes caused by acute systemic inflammation, characterized by high levels of circulating inflammatory markers, as used in most models, relate to the changes that accompany the development of depression in humans with chronic low-grade inflammation. Furthermore, it would be highly informative to determine which specific inflammatory profiles observed in patients with major depressive disorder correspond to the immune responses elicited in a particular experimental models. One potential strategy to enhance the modeling of inflammation-associated depression could involve the chronic peripheral administration of inflammatory mediators designed to recapitulate the specific immune signatures identified in depressive patient.

A primary limitation of most inflammatory models is their acute nature, which contrasts with the chronic course of clinical depression. Investigating the molecular mechanisms underlying LPS tolerance could provide a means to circumvent this limitation, thereby enabling the development of chronic inflammatory models. This is particularly relevant given that, in clinical practice, the therapeutic effects of antidepressants require chronic administration during a prolonged depressive episode. Overall, conventional antidepressants demonstrate efficacy against inflammation-induced depressive-like behavior in rodents. The predictive validity of these models is further supported by the effectiveness of the rapid-acting antidepressant ketamine. Notably, the inconsistent or absent efficacy of acute antidepressant treatment in these models may stem from the fundamental mismatch between an acute experimental challenge and the chronic pathophysiology of depression. Consistent with this, chronic fluoxetine treatment (2 or 3 weeks) attenuates anhedonia-like behavior in a chronic LPS-induced model of depression (201, 202). Therefore, efforts to modify existing models or develop new ones for inflammation-associated depression should prioritize establishing chronic inflammatory states to better satisfy the criteria of face and predictive validity.

Experimental endotoxemia and typhoid vaccination represent the only established models capable of transiently inducing a depressive-like state in humans. A key advantage of these paradigms is their established safety and tolerability. The ability to perform direct comparisons of physiological parameters, sickness behavior, and depressive-like symptoms between human and rodent endotoxemia provides a unique opportunity to critically evaluate the model’s translational strengths and limitations. For instance, such interspecies comparisons raise a critical question regarding optimal dosing: whether lower LPS doses than those typically used in rodent studies would be more appropriate for modeling depressive-like states in rodents (338).

A critical consideration for assessing the face validity of inflammatory models is the alignment between measured parameters and core symptoms of clinical depression. In human experimental models, the administration of inflammatory inducers reliably produces acute mood disturbances, which likely correspond to the low mood in depressed patients. However, this core affective symptom is inherently unmeasurable in rodents. The relevance of common behavioral endpoints in rodents, such as increased immobility in the FST, to specific depressive symptoms in humans remains ambiguous. In contrast, a reduction in preference for sweet solutions may represent a more translationally relevant marker. This parameter is believed to reflect a decrease in the ability to experience pleasure (anhedonia), which is one of the two main symptoms of clinical depression. But sucrose preference test also has several limitations and disadvantages (339). For instance, it has been shown that LPS may suppress sucrose responses by downregulating the expression of Tas1r2 and Tas1r3 transcripts, which encode the T1R2 and T1R3 subunits of the sweet taste receptor. Consequently, a decreased sucrose preference in this context may reflect a sensory deficit rather than, or in addition to, a core anhedonic state, complicating the interpretation of results (340).

The heterogeneity of results observed in LPS-induced neuroinflammation models may be attributed to a range of experimental variables, including the dose, route, and duration of LPS exposure, as well as the species, sex, and age of the subjects (341). The high heterogeneity and limited reproducibility that characterize many rodent studies necessitate a systematic refinement of methodological practices. The following recommendations are proposed to enhance the robustness of preclinical research: 1) Report all methodological details that could influence results; 2) Segregate subjects into phenotypically consistent subgroups (e.g., stress-susceptible vs. resilient) using baseline behavioral assessments before intervention, thereby reducing within-group variance; 3) Validate findings through internal (within-study) and external (independent-lab) replication to ensure robustness.

Beyond investigating the fundamental pathogenesis of depression, there is pressing need for developing novel treatment strategies informed by its pathophysiology. From this perspective, existing inflammatory models in both rodents and humans require further refinement. Such optimization is essential to fully exploit their potential for discovering and validating new, more effective antidepressant therapeutics.

## Glossary

ACTH: Adrenocorticotropic hormone
BBB: Blood-brain barrier
BCG: Bacillus Calmette-Guérin
CCL11: C-C motif chemokine ligand 11
CCL17: C-C motif chemokine ligand 17
CCL3: C-C motif chemokine ligand 3
CCL4: C-C motif chemokine ligand 4
CNS: Central nervous system
CPP: Conditioned place preference
CRP: C-reactive protein
CSF: Cerebrospinal fluid
CUMS: Chronic unpredictable mild stress
CXCL10: C-X-C motif chemokine ligand 10
CXCL-2: C-X-C motif chemokine ligand 2
dACC: Dorsal anterior cingulate cortex
DMT: N,N-dimethyltryptamine
DNA: Deoxyribonucleic Acid
DSM: Diagnostic and Statistical Manual of mental disorders
ELISA: Enzyme-linked immunosorbent assay
EPM: Elevated plus maze test
FABP2/I-FABP: Fatty acid binding protein 2
FGF: Fibroblast growth factor
FST: Forced swim test
GASE: General-Assessment-of-Side-Effects questionnaire
G-CSF: Granulocyte colony-stimulating factor
HBT: Hole-board test
HPAA: Hypothalamic–pituitary–adrenal axis
ICE: Interleukin-1 beta converting enzyme
ICSS: Intracranial self-stimulation
ICV: Intracerebroventricular
IDO: Indoleamine 2,3-dioxygenase
IFN-α: Interferon alpha
IFN-γ: Interferon gamma
IL-1RA: Interleukin-1 receptor antagonist
IL-1β: Interleukin-1β
IL-2: Interleukin-2
IL-3: Interleukin-3
IL-4: Interleukin-4
IL-5: Interleukin-5
IL-6: Interleukin-6
IL-7: Interleukin-7
IL-10: Interleukin-10
IL-12: Interleukin-12
IL-13: Interleukin-13
IL-15: Interleukin-15
IL-16: Interleukin-16
IL-17A: Interleukin-17A
IL-18: Interleukin-18
LBP: LPS-binding protein
LDB: Light–dark box test
LMA: Locomotor activity test
LPS: Lipopolysaccharide
MADRS: Montgomery-Åsberg Depression Rating Scale
MCP-1: Monocyte chemoattractant protein 1
MDBF: German multidimensional mood questionnaire/German version of the POMS
MDD: Major depressive disorder
mRNA: Messenger ribonucleic acid
MWM: Morris water maze test
NMDA: N-Methyl-D-aspartic acid
NSAIDs: Non-steroidal anti-inflammatory drugs
NSFT: Novelty suppressed feeding test
OFT: Open field test
PET: Positron emission tomography
PIGF: Placental growth factor
POMS: Profile of Mood States
SC: Subcutaneous
sCD14: Soluble CD14
SicknessQ: Sickness Questionnaire
sIL-2R: Soluble interleukin 2 receptor
SIT: Social interaction test/social exploration test
SPT: Sucrose preference test/saccharin preference test
SSRI: Selective serotonin reuptake inhibitors
SSS: Stanford Sleepiness Scale
STADI: State Trait Anxiety Depression Inventory
STAI: State-Trait Anxiety Inventory
TNF-α: Tumor necrosis factor alpha
TNF-β: Tumor necrosis factor-beta
TSLP: Thymic stromal lymphopoietin
TSPO: Translocator protein
TST: Tail suspension test
VAS: Visual Analog Scale
VEGF: Vascular endothelial growth factor
VEGF-C: Vascular endothelial growth factor C
VMPFC: Ventromedial prefrontal cortex
WHO: World Health Organization.
